# Methanol‐Regulated Interfacial Polymerization of Fluorinated Polyamide Membranes Enables Rapid Liquid Hydrocarbon Separation

**DOI:** 10.1002/advs.78437

**Published:** 2026-10-27

**Authors:** Xingxiang Li, Mingzheng Yan, Kexin Ma, Jing Li, Yishuo Huang, Wenzhe Liu, Mingrui He, Xianshi Wang, Jun Ma, Zongyao Zhou

**Affiliations:** ^1^ State Key Laboratory of Urban‐rural Water Resources and Environment School of Environment Harbin Institute of Technology Harbin People's Republic of China

**Keywords:** fluorinated polyamide membranes, liquid hydrocarbon separation, methanol‐regulated interfacial polymerization, organic solvent reverse osmosis

## Abstract

Interfacially polymerized polyamide membranes have revolutionized industrial desalination, yet their intrinsically polar networks are poorly suited for molecular separations in nonpolar liquid hydrocarbons. Herein, classical polyamide chemistry is extended to hydrocarbon separations through the incorporation of a hydrophobic trifluoromethyl‐substituted diamine. Methanol was introduced as a cosolvent to overcome the limited aqueous solubility of the fluorinated monomer while regulating interfacial polymerization. Systematic investigation revealed how methanol‐mediated interfacial reaction kinetics and fluorination collectively govern polyamide network formation and membrane structure. The resulting fluorinated polyamide membrane exhibited a 12‐fold increase in toluene permeance and a 51‐fold enhancement in toluene/1,3,5‐triisopropylbenzene (TIPB) separation factor relative to conventional polyamide membranes. The membrane maintained 96%–98% TIPB rejection while sustaining a high average toluene permeance of 0.17 L m^−^^2^ h^−^^1^ bar^−^^1^ during 21 days of continuous operation. Beyond model solute systems, the membrane demonstrated effective molecular discrimination in application‐relevant hydrocarbon mixtures, enabling the fractionation of representative Fischer–Tropsch liquids, rapid enrichment of linear α‐olefins, and efficient separation of light linear/branched alkanes as well as near‐boiling hydrocarbon mixtures. This work establishes fluorinated polyamide membranes as a versatile platform for nonpolar liquid hydrocarbon separations and expands the scope of the highly scalable interfacial polymerization technology beyond aqueous applications.

## Graphical abstract

Through methanol‐regulated interfacial polymerization (IP), bulky trifluoromethyl (‐CF_3_) groups are successfully integrated into polyamide networks. Unlike conventional membranes that suffer from severe solvent‐induced swelling, the fluorinated network sterically resists structural expansion. This anti‐swelling mechanism directly translates to high permeance and precise molecular selectivity for complex liquid hydrocarbon separations.

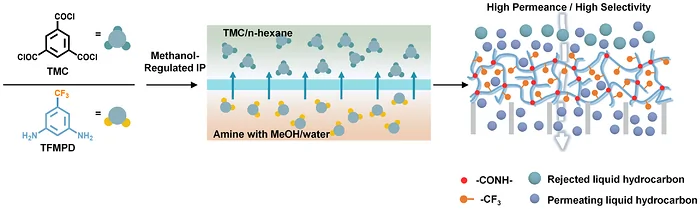

## 1 Introduction

Liquid hydrocarbon separations are central to fuel and chemical production, yet many of these processes still rely on energy‐intensive distillation or operationally complex adsorption [1, 2]. Hydrocarbon processing is shifting from broad boiling‐point fractionation toward precision molecular refining. Consequently, value upgrading increasingly requires separations based on molecular characteristics, including carbon number, degree of unsaturation, and branching [3, 4, 5]. Organic solvent reverse osmosis (OSRO) provides a pressure‐driven, non‐phase‐change route for separating liquid hydrocarbon mixtures [6, 7, 8, 9, 10, 11, 12, 13]. This membrane‐based process exploits differences in molecular size, shape, and membrane‐penetrant interactions. However, realizing this opportunity requires membranes that combine hydrocarbon compatibility, molecular selectivity, chemical stability, and scalable fabrication.

Interfacially polymerized polyamide thin‐film composite (TFC) membranes, the industrial benchmark for water desalination, offer a scalable manufacturing platform [14, 15]. Translating this platform to liquid hydrocarbon separations would leverage existing manufacturing infrastructure rather than require new fabrication paradigms. However, conventional polyamide membranes typically exhibit low permeance and limited molecular selectivity in nonpolar solvents [16, 17]. These limitations arise partly from the inherently hydrophilic nature of polyamide networks, which severely restricts the permeation of nonpolar solvents. In addition, solvent‐induced swelling can expand the polyamide network and reduce molecular selectivity [9, 11, 18].

Fluorinated building blocks can tune surface energetics and polymer‐solvent interactions [7, 19, 20]. The increased hydrophobicity and improved solvent compatibility of fluorinated polyamide facilitate hydrocarbon transport [9, 17, 21]. However, incorporating fluorinated monomers into a defect‐free polyamide selective layer through interfacial polymerization remains challenging. For fluorinated amine monomers used in the aqueous phase, hydrophobic substitution reduces their aqueous solubility. Hydrophobic substitution can also alter monomer partitioning and transport at the aqueous/organic interface [9, 21, 22]. These effects can disrupt monomer transport and network formation during interfacial polymerization, potentially leading to defective or insufficiently crosslinked membranes. To overcome these limitations, cosolvents are often introduced to regulate interfacial polymerization [17, 23]. In a previous study, methanol was employed as a cosolvent to enhance the dissolution of fluorinated monomers in water [17]. Nevertheless, a systematic understanding of methanol‐regulated interfacial polymerization remains lacking. In addition, the effects of fluorination on polyamide network formation and structure have not been comprehensively elucidated.

Here, classical polyamide chemistry is adapted for liquid hydrocarbon separations by coupling fluorinated monomer design with methanol‐regulated interfacial polymerization. The conventional diamine m‐phenylenediamine (MPD) was replaced with 5‐(trifluoromethyl)‐1,3‐phenylenediamine (TFMPD), while trimesoyl chloride (TMC) was retained as the acyl chloride monomer (Figure 1a). The trifluoromethyl‐substituted network is proposed to create a lower‐polarity polyamide environment that promotes hydrocarbon affinity. The bulky ‐CF_3_ groups are also expected to limit solvent‐induced network expansion by providing steric support (Figure 1b,c). Methanol was introduced as a cosolvent to improve TFMPD solubility and regulate interfacial properties. These effects help address the challenges associated with the hydrophobicity and interfacial reactivity of TFMPD. This combined strategy produced fluorinated polyamide thin‐film composite membranes that exhibited hydrocarbon permeance, molecular discrimination, and operational stability.

**FIGURE 1:**
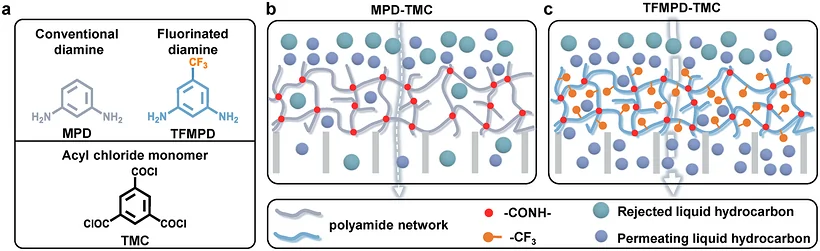
Molecular design of fluorinated polyamide membranes for liquid hydrocarbon separation. (a) Chemical structures of m‐phenylenediamine (MPD), 5‐(trifluoromethyl)‐1,3‐phenylenediamine (TFMPD), and trimesoyl chloride (TMC). (b,c) Schematic representations of the MPD‐TMC and TFMPD‐TMC polyamide networks, highlighting the proposed roles of the ‐CF_3_ groups in regulating hydrocarbon compatibility, chain packing, and solvent‐induced network expansion.

## 2 Results and Discussion

### 2.1 Methanol‐Regulated Interfacial Polymerization

To elucidate the role of methanol, TFMPD‐TMC membranes were prepared using aqueous phases containing 3 to 46 wt.% methanol. These membranes are denoted TFMPD‐TMC‐x, where x is the methanol concentration. The resulting membranes showed distinct morphologies, demonstrating that methanol regulates selective‐layer formation. At moderate methanol concentrations, TFMPD‐TMC membranes formed continuous ridge‐and‐valley, leaf‐like surface features on porous polyacrylonitrile (PAN) supports (Figure 2a,b). Scanning electron microscopy (SEM) and atomic force microscopy (AFM) showed that membranes prepared with 3 to 20 wt.% methanol developed continuous leaf‐like features, with surface roughness reaching 62.7 ± 4.8 nm at 6 wt.% methanol (Figure 2a,b,d). In contrast, the membrane prepared with 46 wt.% methanol exhibited discontinuous ring‐like features and a lower surface roughness of 23.6 ± 0.6 nm. Cross‐sectional SEM images further showed that the apparent selective‐layer thickness increased with increasing methanol concentration (Figure S5).

**FIGURE 2:**
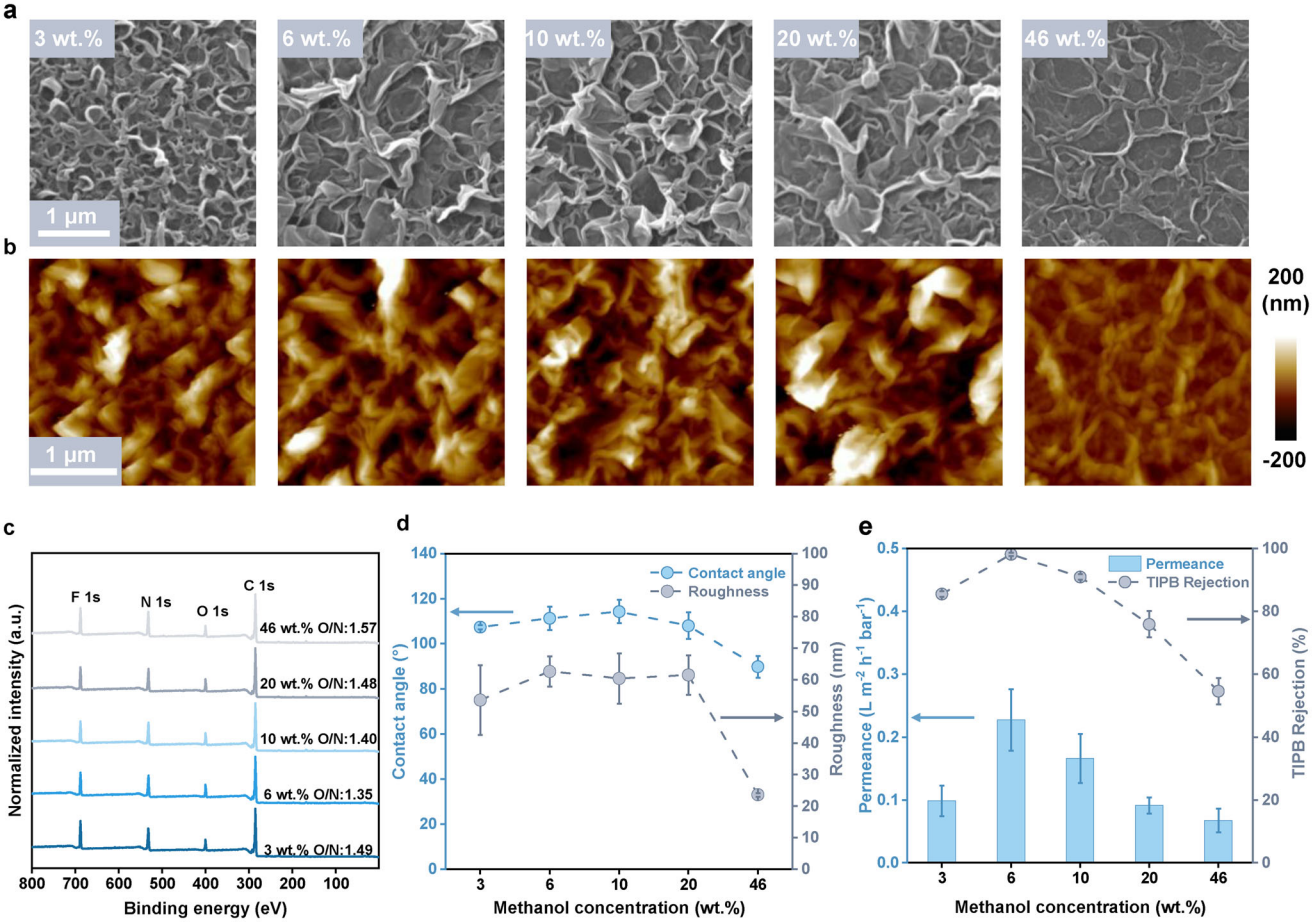
Effects of methanol concentration on the formation and separation performance of TFMPD‐TMC membranes. (a,b) SEM and AFM images of TFMPD‐TMC membranes prepared using aqueous phases containing different methanol concentrations. (c) XPS survey spectra and the corresponding O/N atomic ratios. (d) Water contact angles and AFM‐derived surface roughness as functions of methanol concentration. (e) Toluene permeance and TIPB rejection as functions of methanol concentration, measured using a 99/1 mol% toluene/TIPB feed at room temperature and 20 bar. The data are reported as the mean ± standard deviation for at least three independently prepared membranes.

Chemical characterization confirmed the incorporation of the fluorinated monomer into the polyamide selective layers. Attenuated total reflection Fourier‐transform infrared spectroscopy (ATR‐FTIR) showed characteristic amide bands and C–F stretching vibrations, consistent with fluorinated polyamide formation (Figure S7). High‐resolution x‐ray photoelectron spectroscopy (XPS) peak deconvolution confirmed amide‐related carbonyl/nitrogen species and fluorinated carbon environments associated with TFMPD incorporation (Figures S8–S12). Additionally, scanning electron microscopy‐energy‐dispersive x‐ray spectroscopy (SEM‐EDS) mapping demonstrated a uniform fluorine distribution across the TFMPD‐TMC‐6 membrane surface (Figure S17).

To understand the morphological evolution, the interfacial properties of the aqueous and organic phases were examined. Increasing the methanol concentration decreased the Hansen distance between the aqueous phase and n‐hexane from 44.47 to 34.73 MPa^1/2^ (Table S1), indicating a reduced solubility‐parameter mismatch between the two phases [24]. The surface tension of the aqueous phase also decreased over the same concentration range [25]. Furthermore, methanol may redistribute toward the n‐hexane phase or become enriched near the aqueous/organic interface. This possible redistribution, together with the reduced solubility‐parameter mismatch and lower aqueous‐phase surface tension, may affect monomer transport and interfacial polymerization [23, 26, 27]. At moderate methanol concentrations, these combined effects may facilitate TFMPD transport to the reaction interface. The resulting increase in interfacial monomer availability may promote rapid initial film formation, yielding a highly crumpled surface with increased roughness. At high methanol concentrations, such as 46 wt.%, the increased affinity between the two phases and the possible redistribution of methanol toward the organic phase or interface may broaden the reaction zone and reduce its spatial confinement. This broadened, less‐confined reaction zone may decrease network formation efficiency, ultimately yielding a thicker but smoother and less selective layer [23, 26, 27, 28].

These concentration‐dependent changes in network formation were reflected in the near‐surface composition. TFMPD‐TMC‐6 showed the lowest O/N atomic ratio (1.35) and the highest apparent crosslinking degree (55%) among the series (Figure 2c and Table S2). Conversely, TFMPD‐TMC‐46 exhibited the highest O/N ratio (1.57) and the lowest apparent crosslinking degree (33%), consistent with less efficient network formation. The water contact angles showed a similar trend. Membranes prepared with 3 to 20 wt.% methanol exhibited high contact angles of 107.4° to 114.3° (Figure 2d, Figure S15, and Table S3). In contrast, TFMPD‐TMC‐46 exhibited a notably lower contact angle of 89.8 ± 4.8°, which may be associated with its lower apparent crosslinking degree and reduced surface roughness [29].

Toluene/1,3,5‐triisopropylbenzene (TIPB) separation was used as a benchmark OSRO test to evaluate molecular discrimination [13]. Increasing the methanol concentration from 3 to 6 wt.% increased the toluene permeance from 0.099 ± 0.024 to 0.227 ± 0.049 L m^−^^2^ h^−^^1^ bar^−^^1^. Over the same concentration range, TIPB rejection increased from 85.53% ± 0.93% to 98.11% ± 0.50% (Figure 2e and Table S4). Although TFMPD‐TMC‐3 had a smaller apparent selective‐layer thickness, TEM revealed that its intrinsic PA layer was thicker than those of TFMPD‐TMC‐6 and TFMPD‐TMC‐10, with corresponding thicknesses of 26.0 ± 4.9, 17.0 ± 1.0, and 20.3 ± 3.1 nm, respectively (Figure S6). The resulting increase in transport resistance, together with the less‐developed leaf‐like morphology, likely contributed to the lower toluene permeance of TFMPD‐TMC‐3, whereas its lower TIPB rejection is consistent with its lower apparent crosslinking degree. Further increasing the methanol concentration to 46 wt.% markedly decreased both toluene permeance and TIPB rejection, consistent with the discontinuous and smoother morphology, increased intrinsic PA thickness, and reduced apparent crosslinking degree of TFMPD‐TMC‐46. Overall, 6 wt.% methanol provided the optimal balance among monomer availability, spatial confinement of interfacial polymerization, and selective‐layer integrity.

### 2.2 Effects of Fluorination on Polyamide Network Chemistry and Structure

TFMPD‐TMC‐6 was next compared with conventional MPD‐TMC membranes to determine how trifluoromethyl substitution changes the polyamide network. The high‐resolution XPS C 1s spectrum of TFMPD‐TMC‐6 showed a distinct C–F signal (Figure 3a), confirming the presence of fluorinated functional groups and the difference in chemical composition between the two membranes. When tested with a 99/1 mol% toluene/TIPB feed at room temperature and 20 bar, the conventional MPD‐TMC membranes exhibited a toluene permeance of 0.019 ± 0.001 L m^−^^2^ h^−^^1^ bar^−^^1^ and a TIPB rejection of only 5.94% ± 7.54% (Table S4). These results indicate that the conventional MPD‐TMC polyamide network cannot maintain effective molecular discrimination in liquid hydrocarbon feeds. This difference cannot be attributed solely to selective‐layer defects or discontinuities because both MPD‐TMC and TFMPD‐TMC formed continuous interfacially polymerized layers on PAN supports (Figure S18). Nanomechanical and thermal characterizations further showed comparable mechanical robustness and thermal stability for the two membranes (Figures S19 and S20). Therefore, the effects of the ‐CF_3_ substituent in TFMPD on hydrocarbon affinity, chain packing, and toluene‐induced network expansion were examined.

**FIGURE 3:**
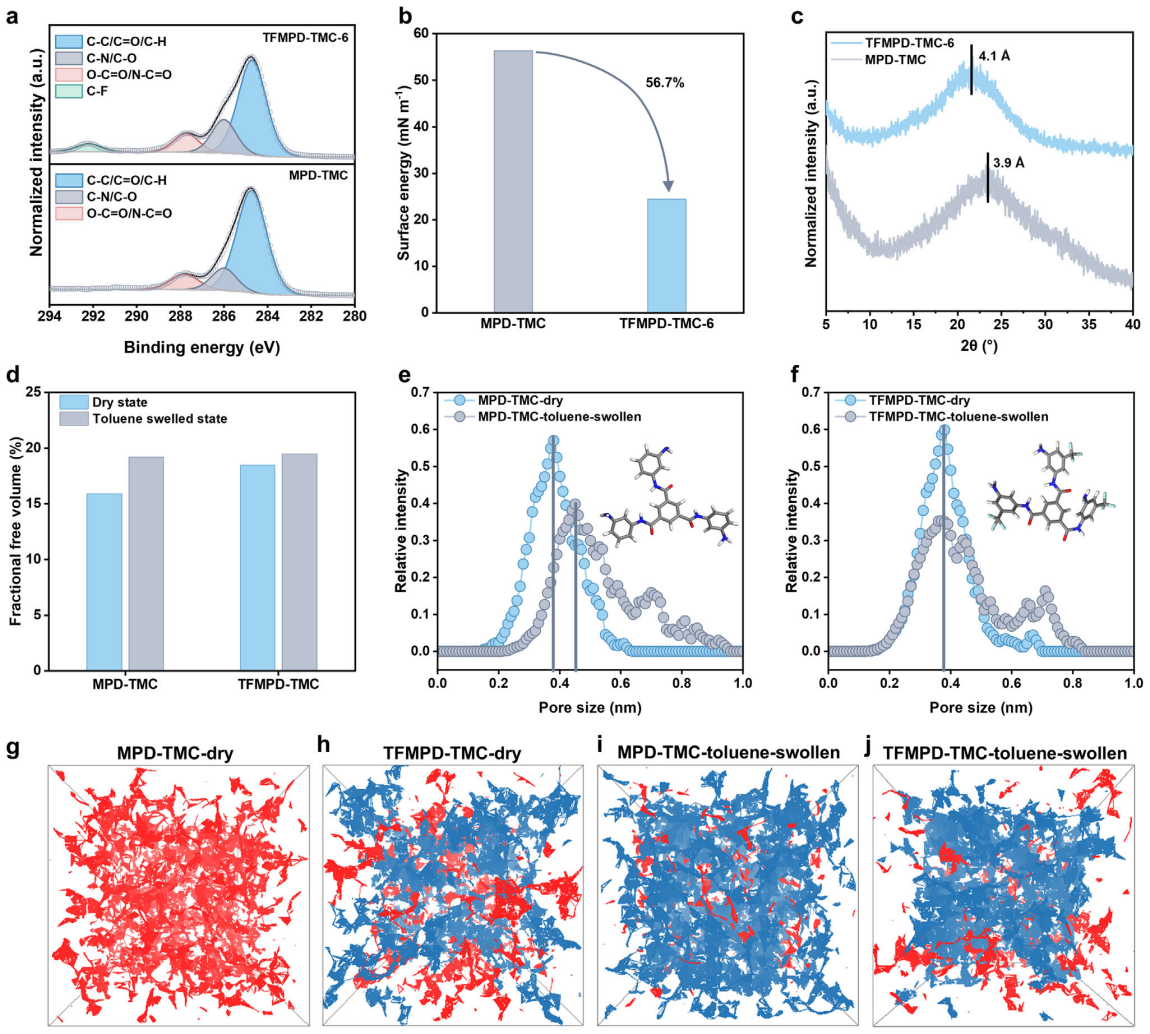
Effects of fluorination on the surface chemistry, chain packing, and structure of polyamide networks. (a) High‐resolution XPS C 1s spectra. (b) Total surface free energy. (c) XRD patterns with average interchain d‐spacings estimated from Bragg's law. (d) Simulated fractional free volume (FFV) in the dry and toluene‐swollen states of the membranes. (e, f) Simulated pore‐size distributions of MPD‐TMC (e) and TFMPD‐TMC (f). (g, h) Dry‐state free‐volume connectivity maps of MPD‐TMC (g) and TFMPD‐TMC (h), calculated using a probe radius of 0.8 Å. (i, j) Toluene‐swollen free‐volume connectivity maps of MPD‐TMC (i) and TFMPD‐TMC (j), calculated using a probe radius of 0.8 Å. The probe radius was used as the connectivity threshold in the Voronoi analysis. Blue and red regions denote interconnected and disconnected voids, respectively.

Surface chemistry governs membrane‐solvent interactions, which are critical for achieving efficient hydrocarbon transport. TFMPD incorporation markedly increased the hydrophobicity of the polyamide layer, as evidenced by an increase in the water contact angle from 56.3° ± 2.5° for MPD‐TMC to 111.3° ± 5.2° for TFMPD‐TMC‐6 (Figure S21 and Table S5). Water, glycerol, and diiodomethane were selected as probe liquids because their complementary polar and nonpolar surface‐tension components enable the surface free‐energy components of the polyamide membranes to be determined using the van Oss–Chaudhury–Good approach (Table S6). Accordingly, the total surface free energy decreased significantly from 56.4 to 24.4 mN m^−^^1^, corresponding to a reduction of 56.7% (Figure 3b and Table S7). This low‐surface‐energy fluorinated environment favors the wetting and partitioning of low‐polarity hydrocarbons, thereby facilitating hydrocarbon transport across the selective layer [19, 20]. The bulky ‐CF_3_ groups also altered chain packing in the polyamide network. X‐ray diffraction (XRD) analysis of the corresponding polyamide powders showed that the average interchain *d*‐spacing increased slightly from 3.9 Å for MPD‐TMC to 4.1 Å for TFMPD‐TMC‐6 (Figure 3c). This modest increase might be attributed to the chain‐expanding effect of the bulky ‐CF_3_ groups in TFMPD rather than to the formation of large open channels within the network [30, 31].

Molecular simulations were performed to compare the free‐volume structures of MPD‐TMC and TFMPD‐TMC membranes in both the dry and solvent‐swollen states (Figure 3d–j). In the dry state, the fractional free volume (FFV) was 15.90% for MPD‐TMC and 18.49% for TFMPD‐TMC, corresponding to a relative increase of 16.3% (Figure 3d and Figure S23). This result indicates greater network microporosity in TFMPD‐TMC, possibly arising from the steric effects of the ‐CF_3_ groups. Despite the higher FFV of TFMPD‐TMC, the two networks exhibited similar average pore sizes of approximately 3.8 Å and comparable pore‐size distributions under dry conditions (Figure 3e,f). In the toluene‐swollen state, however, the two membranes displayed markedly different swelling behavior. MPD‐TMC underwent substantial expansion, with its average pore size increasing from 3.8 to 5.3 Å. By contrast, the average pore size of TFMPD‐TMC increased only from 3.8 to 4.5 Å (Figure 3e,f and Figure S24). Consistently, the simulated volumetric swelling ratio of TFMPD‐TMC was significantly lower than that of MPD‐TMC, with values of 5.35% and 20.69%, respectively (Table S8). This trend was further supported by gravimetric toluene‐uptake measurements using free‐standing PA layers. TFMPD‐TMC‐6 maintained a low uptake throughout the 5‐day immersion period, increasing from 3 wt.% on day 1 to only 7 wt.% on day 5. By contrast, MPD‐TMC exhibited substantially higher and progressively increasing uptake, reaching 35 wt.% on day 1 and 44 wt.% after 5 days (Figure S25). Together, these results indicate that the steric support provided by the ‐CF_3_ groups effectively suppresses solvent‐induced network expansion in TFMPD‐TMC membranes.

Toluene uptake also altered free‐volume connectivity. A Voronoi‐based analysis implemented in Zeo++ was used to evaluate free‐volume connectivity in the dry and toluene‐swollen models. In the dry state, TFMPD‐TMC exhibited a larger fraction of geometrically connected voids than MPD‐TMC membranes (Figure 3g,h and Figures S26 and S27). This difference is likely attributable to the more rigid fluorinated network structure, in which the bulky ‐CF_3_ groups hinder dense chain packing. These results are consistent with the increased interchain *d*‐spacing observed by XRD. After toluene uptake, MPD‐TMC developed extensive interconnected voids, whereas TFMPD‐TMC showed only a limited increase in connectivity (Figure 3i,j and Figures S28 and S29). This result suggests that solvent exposure removes small molecular fragments from the MPD‐TMC network, thereby unblocking numerous previously inaccessible voids [32]. In contrast, TFMPD‐TMC contains substantially fewer removable small molecular fragments, indicating its superior resistance to solvent‐induced structural disruption.

Collectively, these results suggest that fluorination influences the surface chemistry, chain packing, and structure of polyamide. Fluorination markedly decreased the surface energy of the polyamide layer, thereby promoting the preferential partitioning of nonpolar hydrocarbons. The bulky ‐CF_3_ groups suppress pore expansion and mitigate solvent‐induced structural rearrangement, thereby limiting the increase in connected free volume upon solvent uptake. The resulting transport environment is compatible with hydrocarbons yet resistant to unrestricted swelling. Consequently, molecular size and affinity remain effective determinants of selectivity. This balance provides a basis for the enhanced solvent permeance and toluene/TIPB separation of TFMPD‐TMC‐6 and for its broader molecular discrimination in liquid hydrocarbon mixtures.

### 2.3 Benchmarking Separation in OSRO

The benchmarked toluene/TIPB separation performance of the prepared membranes was next compared with that of reported OSRO membranes (Figure 4a and Table S9). The broad performance range of the TFMPD‐TMC series in Figure 4a underscores the tunability of methanol‐regulated interfacial polymerization. In other words, simply by adjusting the methanol‐mediated interfacial polymerization conditions, it is possible to produce fluorinated polyamide membranes with widely tunable permeance and selectivity for nonpolar solvents. Within this series, TFMPD‐TMC‐6 delivered the best balance, with a toluene permeance of 0.227 ± 0.049 L m^−^^2^ h^−^^1^ bar^−^^1^ and a toluene/TIPB separation factor of 55.09. Compared with conventional MPD‐TMC polyamide membranes, which showed a permeance of only 0.019 ± 0.001 L m^−^^2^ h^−^^1^ bar^−^^1^ and a separation factor of 1.07, TFMPD‐TMC‐6 delivered a 12‐fold increase in permeance and a 51‐fold increase in separation factor. Relative to previously reported OSRO membranes, TFMPD‐TMC‐6 resides in the high‐selectivity region of the performance landscape [7, 8, 11, 12, 13, 17, 18, 33, 34, 35, 36, 37, 38]. This comparison supports the use of fluorinated monomer design and methanol‐regulated interfacial polymerization to adapt classical polyamide membranes to liquid hydrocarbon separations.

**FIGURE 4:**
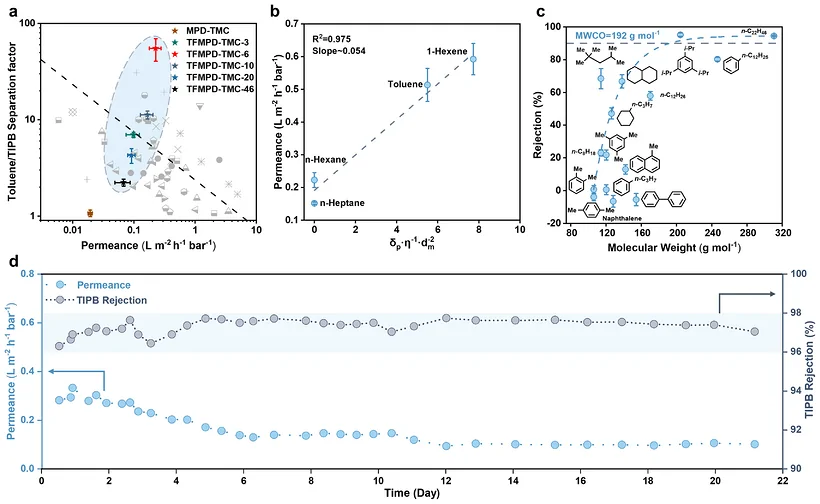
Benchmarking hydrocarbon‐mixture separation performance of TFMPD‐TMC‐6 membranes. (a) Toluene/TIPB separation performance compared with reported OSRO membranes. Note that the literature results were obtained under a range of operating conditions, including pressures of 8–60 bar and TIPB concentrations of 1–10 mol%, as detailed in Table S9. (b) Single‐solvent permeance of representative hydrocarbons through TFMPD‐TMC‐6 plotted against solvent descriptors. η, d_m_, and δ_p_ denote solvent viscosity, estimated molecular diameter, and Hansen polar parameter, respectively. Dashed lines represent linear regression fits. (c) Rejection profile for a diluted 15‐component hydrocarbon mixture in toluene. *Note*: Negative rejection indicates that the solute concentration in the permeate exceeds that in the feed, resulting from the preferential partitioning and transport of these aromatic solutes through the membrane. (d) 21‐day stability test using a 99/1 mol% toluene/TIPB feed. The TFMPD‐TMC‐6 data in panels (a), (b), (c), and (d) were obtained at room temperature and 20 bar.

The single‐solvent permeance of TFMPD‐TMC‐6 membranes followed the order 1‐hexene > toluene > n‐hexane > n‐heptane (Figure 4b). No single solvent descriptor fully accounted for this trend. Instead, viscosity, molecular size, and solvent polarity jointly influenced solvent transport (Figure 4b and Figure S30). Hydrocarbon permeation through TFMPD‐TMC‐6 therefore reflects coupled contributions from molecular diffusion, solvent partitioning, and membrane‐solvent interactions, as commonly observed in polyamide membranes [16, 39]. Molecular discrimination was then evaluated using a diluted 15‐component hydrocarbon mixture in toluene. The solutes covered molecular weights from 106 to 310 g mol^−^^1^ and included linear, branched, cyclic, and aromatic hydrocarbons (Figure 4c and Table S13). TFMPD‐TMC‐6 exhibited an apparent hydrocarbon molecular‐weight cutoff of approximately 192 g mol^−^^1^, falling within the broad apparent MWCO range reported for OSRO membranes [40]. Rejection was not governed by molecular weight alone; molecular geometry and solute‐membrane interactions also contributed. Several aromatic solutes, including naphthalene and biphenyl, showed near‐zero or negative rejection, consistent with favorable partitioning and transport through the fluorinated aromatic polyamide matrix (Figure S31) [12, 13]. It is worth noting that the membrane could also differentiate between molecules of identical molecular weight but different kinetic diameters. Isooctane was rejected at 68.45% ± 6.16%, whereas n‐octane was rejected at only 23.11% ± 1.95%, despite both having a molecular weight of 114.22 g mol^−^^1^. TFMPD‐TMC‐6 therefore still exhibited notable separation performance between the two isomers (Figure 4c). This selectivity, arising from the subtle difference in kinetic diameter (0.43 vs. 0.62 nm) [41], is attributed to the minimal swelling of the membrane.

Operational stability was assessed using the 99/1 mol% toluene/TIPB feed. During 21 days of operation, TIPB rejection remained between 96% and 98% (Figure 4d and Table S10), confirming retention of molecular selectivity under prolonged hydrocarbon exposure. Toluene permeance decreased during the early stage of operation and then stabilized at approximately 0.10 L m^−^^2^ h^−^^1^ bar^−^^1^ after about 12 days. During the continuous test, toluene preferentially permeated through the membrane, whereas TIPB was strongly retained. Consequently, the estimated TIPB concentration in the remaining feed increased progressively, which may have increased the transmembrane osmotic‐pressure difference and reduced the effective driving force for toluene transport [38]. A possible contribution from limited pressure‐induced compaction of the composite membrane cannot be excluded [16, 42]. Nevertheless, the stable TIPB rejection indicates that the selective layer retained its molecular‐discrimination ability throughout the test. The membrane maintained an average permeance of 0.17 L m^−^^2^ h^−^^1^ bar^−^^1^ over the 3 weeks of testing. This stable performance even exceeds the instantaneous toluene permeance of many reported OSRO membranes, as shown in Figure 4a, highlighting the rapid nonpolar‐solvent separation capability of the developed fluorinated polyamide membranes.

### 2.4 Separation of Liquid Aliphatic Mixtures

TFMPD‐TMC‐6 was further evaluated using liquid aliphatic mixtures that require molecular discrimination. These mixtures were selected to represent both broad‐cut prefractionation of synthetic hydrocarbon liquids and targeted downstream purification tasks, including olefin enrichment and alkane‐isomer discrimination. These tests examined whether the size‐ and affinity‐dependent transport observed in benchmark hydrocarbon separations could be translated to application‐relevant fractionation and purification. First, a representative broad‐cut liquid feed inspired by Fischer–Tropsch (FT) products, which commonly contain linear hydrocarbons over a wide carbon‐number range, was used [43, 44]. Because FT product distributions vary with the catalyst and reaction conditions [44, 45], a simplified composition was adopted rather than reproducing a specific process stream. The feed contained linear alkanes from C6 to C22 and approximately 4 wt.% 1‐hexene as a representative light α‐olefin (Table S14).

Figure 5a shows the permeate‐to‐retentate concentration ratio, *C_P_/C_R_*, for each component in the FT mixture. The membrane produced clear carbon‐number‐dependent fractionation across the n‐alkane series. Short‐chain components were enriched in the permeate, whereas longer‐chain alkanes were preferentially retained. The *C_P_/C_R_* values of 1‐hexene and n‐hexane (n‐C6) reached 1.47 ± 0.02 and 1.31 ± 0.01, respectively. In contrast, n‐docosane (n‐C22) showed a very low *C_P_/C_R_* value of 0.19 ± 0.04. Among the linear alkanes, *C_P_/C_R_* decreased monotonically with increasing carbon number, indicating increasing transport resistance with chain length. 1‐Hexene showed a higher *C_P_/C_R_* than n‐hexane despite their similar carbon numbers, consistent with its higher single‐solvent permeance. This difference suggests that unsaturation also affects partitioning or transport in the fluorinated polyamide network. Fractionation in this broad‐cut mixture is therefore governed mainly by chain‐length‐sensitive transport, with an additional contribution from unsaturation‐dependent interactions.

**FIGURE 5:**
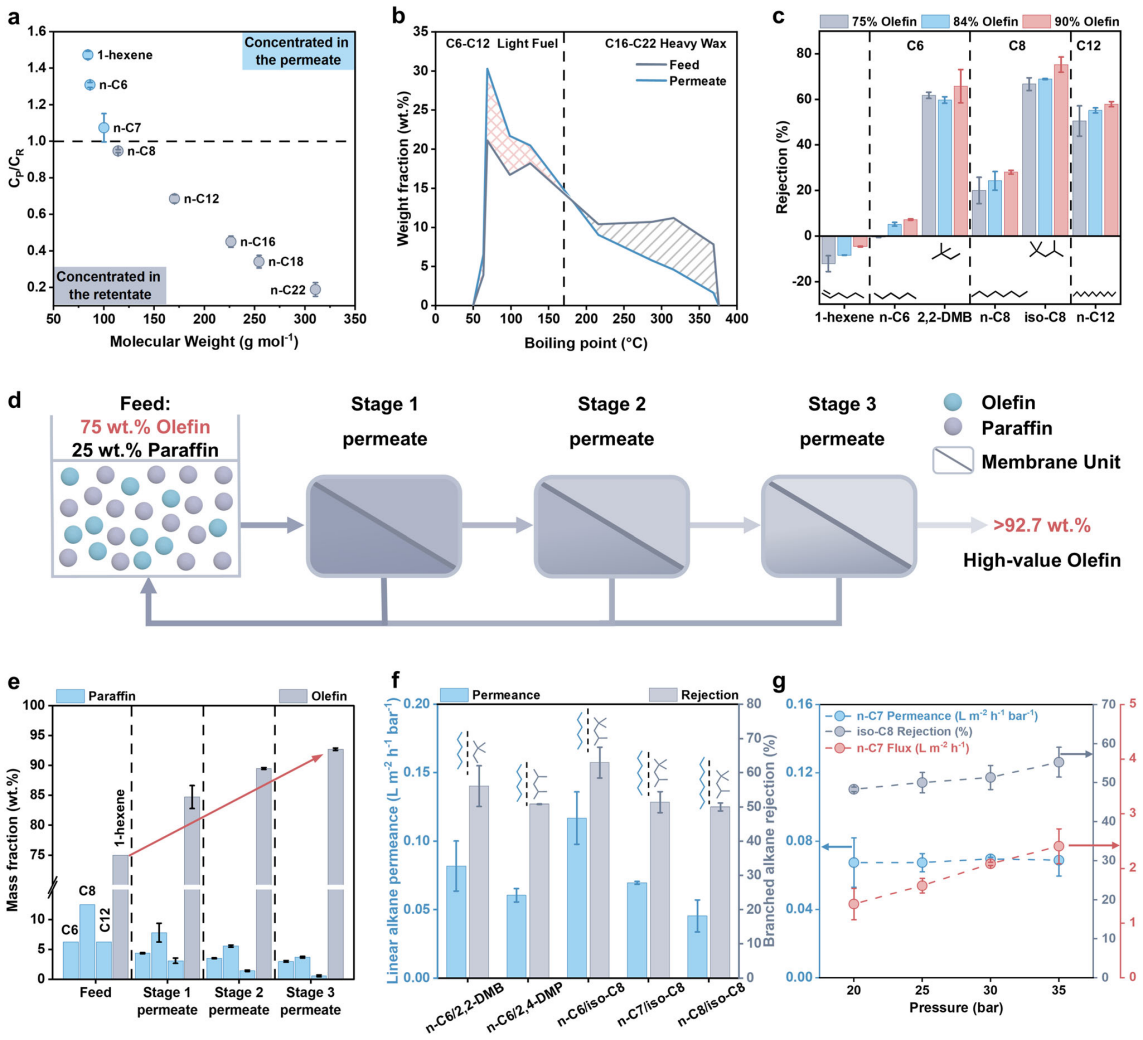
Liquid aliphatic mixture separation performance of TFMPD‐TMC‐6 membranes. (a) Permeate‐to‐retentate concentration ratio (CP/CR) for individual components after single‐pass fractionation of a representative FT product mixture. (b) Boiling‐point distributions of the feed and permeate from the FT fractionation experiment. (c) Olefin/paraffin separation performance for TFMPD‐TMC‐6. The initial feed contained 75 wt.% 1‐hexene, 3.125 wt.% n‐hexane, 3.125 wt.% 2,2‐dimethylbutane, 8.75 wt.% n‐octane, 3.75 wt.% isooctane, and 6.25 wt.% n‐dodecane. Feeds with olefin contents of 84 and 90 wt.% were prepared by increasing the olefin fraction while keeping the relative paraffin composition constant (Table S16). (d) Schematic of the three‐stage membrane cascade for linear α‐olefin enrichment. (e) Evolution of 1‐hexene purity over the three‐stage cascade, starting from a feed containing 75 wt.% 1‐hexene. (f) Branched alkane rejection and feed permeance for five binary 99/1 mol% linear/branched alkane mixtures. (g) Pressure‐dependent separation performance for a 99/1 mol% near‐boiling n‐heptane/isooctane mixture. All measurements were conducted at room temperature. The experiments in panel (a) were performed at 35 bar, those in panels (c), (e), and (f) at 30 bar, and those in panel (g) at the indicated pressures.

The fractionation effect was further visualized through the boiling‐point distributions of the feed and permeate streams (Figure 5b). The C6‐C12 hydrocarbons were grouped as the light‐fuel fraction, while the C16‐C22 hydrocarbons were categorized as the heavier wax/lubricant‐range fraction. After permeation, the light‐fuel content increased from 70.3 wt.% in the feed to 88.0 wt.% in the permeate, whereas the C16‐C22 hydrocarbons were preferentially retained. This compositional shift produced a light‐hydrocarbon‐enriched permeate that may be suitable for downstream upgrading into light fuels or olefins [46, 47]. The corresponding retentate was enriched in heavier paraffins, which could serve as precursors for wax‐ or lubricant‐range products [44, 48]. For liquid‐phase aliphatic separations, the FRPAA membrane represents the current state of the art, with a reported permeance of approximately 0.01 L m^−^^2^ h^−^^1^ bar^−^^1^ [7]. Under comparable testing conditions, TFMPD‐TMC‐6 exhibited roughly twice this permeance (Table S15), which can be attributed to its thinner selective layer and slightly larger free volume, resulting from the tunable methanol‐regulated interfacial polymerization. Nevertheless, compared with the permeance observed for single solvents, the overall permeance for this complex feed was relatively low, due to the higher transport resistance of concentrated multicomponent hydrocarbon mixtures.

Liquid linear α‐olefins (LAOs) are high‐value feedstocks for polyolefins, surfactants, plasticizers, and poly‐α‐olefin base oils [49, 50, 51]. Separating LAOs from compositionally similar paraffins is important for downstream purification of olefin‐containing hydrocarbon streams, including FT‐derived light fractions, but remains challenging [52]. 1‐Hexene was used as a representative light LAO to evaluate olefin enrichment from liquid olefin/paraffin mixtures. The initial multicomponent feed contained 75 wt.% 1‐hexene and 25 wt.% C6‐C12 alkane impurities, including linear and branched alkanes (Table S16). TFMPD‐TMC‐6 preferentially transported 1‐hexene while rejecting bulkier branched alkanes and heavier linear alkanes (Figure 5c and Table S16). For the 75 wt.% 1‐hexene feed, 2,2‐dimethylbutane (2,2‐DMB) and isooctane (iso‐C8) rejections were 61.80% ± 1.40% and 66.71% ± 2.81%, respectively, and n‐dodecane (n‐C12) rejection was 50.54% ± 6.69%. By contrast, 1‐hexene showed negative rejection (−12.17% ± 3.53%), corresponding to enrichment in the permeate. Separation of 1‐hexene from n‐hexane (n‐C6) and n‐octane (n‐C8) proved more challenging, consistent with the similar size and shape of these three components. When the 1‐hexene content in the feed was increased to 90 wt.% while maintaining the relative impurity ratios, paraffin rejection generally increased. For example, isooctane rejection reached 75.29% ± 3.38%. These results showed preferential 1‐hexene transport and rejection of branched or heavier alkane impurities.

Subsequent tests examined whether this single‐pass preference could be amplified by staged operation. In a three‐stage pressure‐driven cascade, the permeate from each stage was used as the feed for the next stage (Figure 5d). The 1‐hexene content increased from 75.0 wt.% in the initial feed to 84.72 ± 1.92 wt.% after the first stage, 89.48 ± 0.15 wt.% after the second stage, and 92.71 ± 0.19 wt.% after the third stage (Figure 5e). The permeance increased from 0.074 ± 0.007 to 0.149 ± 0.009 L m^−^^2^ h^−^^1^ bar^−^^1^ across the cascade (Table S17), consistent with the increasing fraction of the more permeable 1‐hexene in later‐stage feeds. Staged operation therefore amplified the recovery and enabled stepwise LAO enrichment.

The most stringent test involved precise separation of light linear and branched alkanes. This separation is relevant to gasoline upgrading because branched alkanes have higher research octane numbers (RONs) than their linear counterparts [53]. However, linear and branched alkanes often have similar polarity and volatility, making conventional separations challenging [54, 55]. For example, n‐heptane and isooctane differ in boiling point by only approximately 1°C [56]. Membrane separations offer an opportunity to fractionate these mixtures through molecular size discrimination.

At room temperature and 30 bar, TFMPD‐TMC‐6 preferentially rejected branched alkanes from all five C6‐C8 binary linear/branched alkane feeds tested (Figure 5f and Table S18). The C6 isomeric pair n‐hexane/2,2‐dimethylbutane represents a particularly challenging separation because the two molecules have identical carbon numbers and molecular weights but different degrees of branching. Reported liquid‐phase OSRO membranes illustrate this difficulty [7]. Thermally crosslinked Matrimid rejected only 3.4% of 2,2‐DMB, whereas fluorine‐rich FRPAA‐1 achieved 60.6% rejection at 80 bar with a permeance of 0.036 L m^−^^2^ h^−^^1^ bar^−^^1^. TFMPD‐TMC‐6 delivered a comparable 2,2‐DMB rejection of 56.09% ± 5.99% at a lower pressure of 30 bar. It also maintained a permeance of 0.082 ± 0.018 L m^−^^2^ h^−^^1^ bar^−^^1^, more than twice that reported for FRPAA‐1. Similar precise discrimination was observed for the other feeds. Isooctane rejection reached 49.97 ± 1.15% for the n‐octane/isooctane (n‐C8/iso‐C8) isomeric pair and 51.36 ± 3.08% for the near‐boiling n‐heptane/isooctane (n‐C7/iso‐C8) mixture. The highest branched‐alkane rejection was observed for n‐hexane/isooctane (n‐C6/iso‐C8), reaching 62.95 ± 4.51%, consistent with the larger difference in molecular size and branching. The rejection of 2,4‐dimethylpentane in the n‐hexane/2,4‐dimethylpentane (n‐C6/2,4‐DMP) feed was 50.82 ± 0.05%. Overall, branched alkane rejections remained in the range of 50–63% across all five binary feeds, indicating that TFMPD‐TMC‐6 retains good separation performance even in challenging systems where differences in molecular size, molecular weight, shape, and membrane affinity are minimal.

Pressure‐dependent transport was further evaluated using the near‐boiling n‐heptane/isooctane mixture. As the applied pressure increased from 20 to 35 bar, the flux rose from 1.35 ± 0.29 to 2.41 ± 0.32 L m^−^^2^ h^−^^1^, while the permeance remained nearly constant at approximately 0.07 L m^−^^2^ h^−^^1^ bar^−^^1^ (Figure 5g and Table S19). Meanwhile, isooctane rejection increased from 48.29% ± 0.60% to 55.27% ± 3.83%, indicating preferential n‐heptane transport while the bulkier branched isooctane remained restricted. This pressure response is consistent with solution‐diffusion transport through selective regions rather than defect‐mediated convective leakage [38]. Consequently, higher operating pressures led to increased flux while maintaining separation selectivity.

## 3 Conclusion

Classical interfacial polymerization can be repurposed for liquid hydrocarbon separation by coupling fluorinated monomer design with methanol‐regulated film formation. The trifluoromethyl‐substituted network provides a low‐surface‐energy, hydrocarbon‐compatible environment that suppresses solvent‐induced expansion. Methanol acts as an interfacial regulator, overcoming the limited aqueous solubility of the fluorinated monomer and optimizing selective‐layer formation. The optimized TFMPD‐TMC‐6 membrane increased the toluene permeance by 12‐fold and the toluene/TIPB separation factor by 51‐fold over conventional polyamide membranes while maintaining hydrocarbon permeance and 21‐day operational stability. This intrinsic molecular discrimination translates to application‐relevant complex mixtures, enabling Fischer–Tropsch liquid fractionation, staged enrichment of linear α‐olefins, precise discrimination between linear and branched alkanes, and efficient separation of near‐boiling hydrocarbon mixtures. By extending the scalable polyamide TFC manufacturing platform to liquid hydrocarbon separation, this strategy provides a practical route toward pressure‐driven and non‐phase‐change molecular refining in the petrochemical industry.

## Author Contributions

**Xingxiang Li**: conceptualization, methodology, investigation, validation, writing – original draft, data curation, formal analysis, visualization. **Mingzheng Yan**: conceptualization, methodology, formal analysis, visualization. **Kexin Ma**: methodology. **Jing Li**: formal analysis. **Yishuo Huang**: methodology. **Wenzhe Liu**: investigation, formal analysis. **Mingrui He**: methodology. **Xianshi Wang**: methodology. **Jun Ma**: funding acquisition, resources. **Zongyao Zhou**: supervision, resources, writing – review and editing, funding acquisition, conceptualization.

## Conflicts of Interest

The authors declare no conflicts of interest.

## Supporting information




**Supporting File**: advs78437‐sup‐0001‐SuppMat.docx.

## Data Availability

The data that supports the findings of this study are available in the supplementary material of this article.
