# Supplementary material for: Methanol‐Regulated Interfacial Polymerization of Fluorinated Polyamide Membranes Enables Rapid Liquid Hydrocarbon Separation
Source: Adv Sci (Weinh). 2026 Oct 27:e78437. Online ahead of print. doi: 10.1002/advs.78437 (PMC13711143; doi:10.1002/advs.78437)
Supplement: Supplementary file 1 [file ADVS-9999-e78437-s001.docx]

Supplementary Information

Methanol-Regulated Interfacial Polymerization of Fluorinated Polyamide Membranes Enables Rapid Liquid Hydrocarbon Separation

Xingxiang Li^[a]^, Mingzheng Yan^[a]^, Kexin Ma^[a]^, Jing Li^[a]^, Yishuo Huang^[a]^, Wenzhe Liu^[a]^, Mingrui He^[a]^, Xianshi Wang^[a]^, Jun Ma^[a]^, Zongyao Zhou*^[a]^

[a]	X. Li, M. Yan, K. Ma, J. Li, Y. Huang, W. Liu, M. He, X. Wang, J. Ma, Z. Zhou
State Key Laboratory of Urban-rural Water Resources and Environment
School of Environment, Harbin Institute of Technology
Harbin 150090, P. R. China
E-mail: zongyao.zhou@hit.edu.cn

# Materials

m-Phenylenediamine (MPD, >99.5%), trimesoyl chloride (TMC, >98%), and 5-(trifluoromethyl)-1,3-phenylenediamine (TFMPD, >98%) were purchased from Shanghai Aladdin Bio-Chem Technology Co., Ltd. Isopropanol (IPA), N,N-Dimethylacetamide (DMAc) and ethanol were obtained from Sinopharm Chemical Reagent Co., Ltd. Methanol was purchased from Shanghai Macklin Biochemical Technology Co., Ltd. Flat-sheet polyacrylonitrile (PAN) ultrafiltration membranes (MWCO = 50 kDa) were obtained from Beijing Rising Sun Membrane Co., Ltd. and used as the porous support for interfacial polymerization. Ultrapure water was supplied by a PU-UV laboratory water purification system from Hyper Pure X (Shanghai). Glycerol (Gly, >99.5%) and diiodomethane (CH_2_I_2_, >99.5%), used for contact-angle measurements, were obtained from Shanghai Macklin Biochemical Technology Co., Ltd.

Unless otherwise specified, all hydrocarbons used for membrane-separation experiments, including 1-hexene, n-hexane, n-heptane, n-octane, n-dodecane, n-hexadecane, n-octadecane, n-docosane, 2,2-dimethylbutane, 2,4-dimethylpentane, isooctane, toluene, p-xylene, o-xylene, propylcyclohexane, propylbenzene, mesitylene, decalin, naphthalene, 1-methylnaphthalene, 1,3,5-triisopropylbenzene (TIPB), biphenyl, and dodecylbenzene, were purchased from Shanghai Macklin Biochemical Technology Co., Ltd. and used without further purification.

# Preparation of thin-film composite (TFC) membranes

Thin-film composite (TFC) membranes were fabricated on porous PAN substrates by interfacial polymerization between diamine monomers in the aqueous phase and TMC in the organic phase. For the MPD-TMC system, the aqueous phase consisted of 2 wt.% MPD in water. For the TFMPD-TMC system, TFMPD was used as the aqueous-phase diamine monomer. Because TFMPD exhibits limited solubility in water but dissolves readily in methanol, it was first dissolved in methanol and then diluted with water to prepare the aqueous phase. In all cases, the TFMPD concentration was fixed at 2 wt.%, while the methanol concentration was varied to 3, 6, 10, 20, or 46 wt.% relative to the total mass of the aqueous phase. The organic phase consisted of 0.15 wt.% TMC in n-hexane.

To fabricate the membranes, the aqueous phase was applied to the PAN substrate and allowed to remain in contact for 5 min at room temperature. Excess aqueous phase was removed using a rubber roller. The organic phase was then brought into contact with the substrate for 2 min to initiate interfacial polymerization. After removal of the excess organic phase, the resulting membrane was thermally cured in an oven at 100 °C for 5 min, immersed in IPA to remove residual unreacted species, and stored in IPA before characterization and permeation measurements. These membranes are denoted TFMPD-TMC-x, where x is the methanol concentration.

# Preparation of polyamide powders

Polyamide powders were prepared using the same monomer compositions and solution formulations as those used for membrane fabrication, except that the reaction was carried out in bulk rather than on a porous substrate. Polyamide powder formation was initiated by combining 50 mL of the aqueous solution with 150 mL of the organic solution in a beaker, followed by magnetic stirring at 300 rpm for 30 min. The resulting polymer was collected by vacuum filtration, washed three times each with 500 mL of n-hexane, ethanol, and water, and dried in a vacuum oven at 120 °C for 12 h, following a previously reported procedure with minor modifications.^[1]^ The final mass of the collected polyamide powders was approximately 0.14 g for MPD-TMC and 0.17 g for TFMPD-TMC-6.

# Preparation of free-standing polyamide nanofilms

Free-standing polyamide nanofilms were prepared by dissolving the PAN support layer of the as-prepared TFC membranes in dimethylacetamide (DMAc). Specifically, the dried polyamide/PAN composite membranes were immersed in DMAc until the PAN support layer was completely dissolved, causing the polyamide selective layer to detach from the substrate and float on the solution surface. The free-standing polyamide film was then carefully transferred to deionized water using a 300-mesh nylon screen and soaked to remove residual DMAc. Finally, the film was collected onto a silicon wafer and dried at 60 °C for subsequent thickness characterization^[2]^.

# Characterization

Scanning electron microscopy (SEM)**.** Membrane surface and cross-sectional morphologies were examined using a field-emission scanning electron microscope (SEM; Sigma 500, ZEISS, Germany). Before imaging, the samples were sputter-coated with a thin platinum layer for 120 s to minimize surface charging. SEM images were acquired at an accelerating voltage of 10 kV.

Atomic force microscopy (AFM). Surface topography, roughness, and nanomechanical properties were characterized by atomic force microscopy (AFM; NanoWizard 4, Bruker, Germany) operated in both tapping mode and quantitative nanomechanical mapping (QNM) mode. Topography images were acquired in tapping mode over areas of 3 × 3 µm or 5 × 5 µm, depending on the measurement. Surface roughness values were calculated as the arithmetic mean deviation from at least three independently selected areas (3 × 3 µm each), unless otherwise noted. Young's modulus maps were obtained in QNM mode over 5 × 5 µm scan areas. AFM data were processed and analyzed using NanoScope Analysis 3.0.

Transmission electron microscopy (TEM). The freestanding PA film floating on the water surface was transferred onto a copper grid and air-dried. The film morphology was then examined using a transmission electron microscope (TEM; Talos F200i, Thermo Fisher Scientific, USA) operated at an accelerating voltage of 200 kV.

Fourier transform infrared spectroscopy (FTIR). Chemical functionalities were identified by Fourier transform infrared spectroscopy (FTIR; Frontier, PerkinElmer, USA) in attenuated total reflection (ATR) mode. Each spectrum was collected over the range of 600–4000 cm^-1^ with 32 scans at a resolution of 4 cm^-1^.

X-ray photoelectron spectroscopy (XPS). Surface elemental compositions and chemical states were analyzed by X-ray photoelectron spectroscopy (XPS; K-Alpha, Thermo Fisher Scientific, USA) using a monochromatic Al Kα X-ray source (*hν* = 1486.68 eV). The X-ray source was operated at 12 kV and 6 mA. The base pressure in the analysis chamber was approximately 5×10^-9^ mbar. High-resolution spectra of C 1s, N 1s, O 1s, and F 1s were collected at a pass energy of 50 eV with a step size of 0.1 eV. All binding energies were charge-corrected by setting the hydrocarbon C 1s peak to 284.8 eV. The spectra were analyzed using Advantage software.

X-ray diffraction (XRD). The average interchain d-spacing of polyamide powders was estimated using an X-ray diffractometer (D8 Advance, Bruker, Germany) equipped with a Cu Kα radiation source (λ = 0.15406 nm), operated at 40 kV and 40 mA. Diffraction patterns were recorded over a 2θ range of 5–40° with a step size of 0.02°.

Thermogravimetric analysis (TGA). The thermal stability of the polyamide powders was evaluated by thermogravimetric analysis (TGA) using a STA200 thermal analyzer (HITACHI, Japan) under a nitrogen atmosphere from 30 to 800 °C at a heating rate of 10 °C min⁻¹.

Surface contact-angle measurements. Static contact angles of water, glycerol, and diiodomethane on membrane surfaces were measured at room temperature using a contact-angle goniometer (HARKE-SPCA, Shanghai Fangrui Instrument Co., Ltd., China). For each sample, at least six measurements were taken at randomly selected locations, and the results are reported as the average ± standard deviation.

Energy-dispersive X-ray spectroscopy (EDS). Elemental distributions on the membrane surfaces were characterized by energy-dispersive X-ray spectroscopy (EDS) mapping attached to the SEM instrument. EDS elemental maps were collected from representative membrane surface regions to visualize the spatial distributions of C, N, O, and F.

# Pressure-driven permeation and separation measurements

The pressure-driven permeation and separation performance of the as-prepared membranes was evaluated using a stainless-steel stirred dead-end filtration cell. The effective membrane area was 0.196 cm². A dead-end configuration was used here as a laboratory-scale screening platform for membrane performance evaluation, while continuous stirring was applied to reduce concentration polarization. Unless otherwise specified, at least three independently prepared membranes were tested for each condition, and the results are reported as mean ± standard deviation.

Before each test, the membrane was equilibrated with the corresponding solvent or feed under the applied pressure until a stable flux was reached. Unless otherwise specified, the reported permeance values were obtained after quasi-steady-state conditions had been established.

The solvent permeance *P* was calculated as

$$P=\frac{V}{A\Delta pt} (1)$$

where *V* is the permeate volume, *A* is the effective membrane area, Δ*p* is the applied pressure, and *t* is the permeation time.

The solute rejection 𝑅 was calculated as

$$R=\left( 1-\frac{C_{p}}{C_{f}} \right)\times100\% (2)$$

where *C_p_* and *C_f_* are the solute concentrations in the permeate and feed, respectively. Negative rejection indicates that the solute concentration in the permeate exceeded that in the feed, consistent with preferential partitioning and/or transport through the membrane.

For binary mixtures, the separation factor of component *A* over component *B* was calculated as

$$\alpha_{A/B}=\frac{C_{A,P}/C_{A,R}}{C_{B,P}/C_{B,R}} (3)$$

where *C_A,P_* and *C_A,R_* are the concentrations of component 𝐴 in the permeate and retentate, respectively, and *C_B,P_* and *C_B,R_* are defined analogously for component *B*.

The concentrations of hydrocarbons in the feed, permeate, and retentate were determined by gas chromatography (GC). To minimize concentration polarization, all permeation tests were performed under continuous stirring.

# Surface free-energy analysis

The surface free energy of the polyamide membranes was calculated from the contact angles of three probe liquids. The equilibrium contact angle of a liquid on a solid surface is described by Young’s equation^[3, 4]^:

$$\gamma_{SV}=\gamma_{SL}+\gamma_{LV}cos\theta(4)$$

where $\gamma_{SV}$ , $\gamma_{SL}$ and $\gamma_{LV}$ are the solid-vapor, solid-liquid, and liquid-vapor interfacial tensions, respectively and 𝜃 is the equilibrium contact angle.

To determine the surface free-energy components of the polyamide membranes, the van Oss–Chaudhury–Good approach was used^[5, 6]^. The total surface free energy of the membrane ($\gamma_{s}^{total}$ ) can be expressed as the sum of the Lifshitz-van der Waals component ($\gamma_{s}^{LW}$) and the acid-base component ($\gamma_{s}^{AB}$)

$$\gamma_{s}^{total}=\gamma_{s}^{LW}+\gamma_{s}^{AB}=\gamma_{s}^{LW}+2\sqrt{\gamma_{s}^{+}\gamma_{s}^{-}} (5)$$

where $\gamma_{s}^{+}$and $\gamma_{s}^{-}$ are the electron-acceptor and electron-donor parameters of the membrane surface, respectively.

For a probe liquid *l*, the relationship between the contact angle (θ) and the surface-energy components is given by the van Oss–Chaudhury–Good equation:

$$(1+cos\theta)\gamma_{l}^{total}=2(\sqrt{\gamma_{s}^{LW}\gamma_{l}^{LW}}+\sqrt{\gamma_{s}^{+}\gamma_{l}^{-}}+\sqrt{\gamma_{s}^{-}\gamma_{l}^{+}}) (6)$$

where$\gamma_{l}^{total}$ is the total surface tension of the probe liquid, and $\gamma_{l}^{LW}$, $\gamma_{l}^{+}$ and$\gamma_{l}^{-}$are its Lifshitz-van der Waals, electron-acceptor, and electron-donor components, respectively. The surface-tension parameters of the probe liquids used in this work are listed in Table S6.

# Hansen solubility parameter analysis

The Hansen solubility parameter (HSP) framework describes intermolecular interactions using three components: dispersion interactions (*δ_d_*), polar interactions (*δ_p_*), and hydrogen-bonding interactions (*δ_h_*)^[7]^. The total solubility parameter is given by

$$\delta=\sqrt{{{\delta_{d}^{2}+\delta}_{p}^{2}+\delta}_{h}^{2}} (7)$$

where all parameters are expressed in units of MPa^1/2^.

For solvent mixtures, the HSP components were estimated using a volume-fraction-weighted mixing rule^[8, 9]^

$$\delta_{d,mix}=\sum_{i} \phi_{i}\delta_{d,i} (8)$$

$$\delta_{p,mix}=\sum_{i} \phi_{i}\delta_{p,i} (9)$$

$$\delta_{h,mix}=\sum_{i} \phi_{i}\delta_{h,i} (10)$$

where *ϕ_i_* is the volume fraction of component *i* and $\sum_{i} \phi_{i}=1$. The volume fraction of each component was calculated from its mass and density

$$\phi_{i}=\frac{m_{i}/\rho_{i}}{\sum_{j} m_{j}/\rho_{j}} (11)$$

where *m_i_* and *ρ_i_* are the mass and density of component *i*, respectively.

The total solubility parameter of the mixture was then calculated as

$$\delta_{mix}=\sqrt{{{\delta_{d,mix}^{2}+\delta}_{p,mix}^{2}+\delta}_{h,mix}^{2}} (12)$$

To compare the relative affinity between different substances, the Hansen distance (*R_a_*) between substances 1 and 2 was calculated as

$$R_{a}=\sqrt{4\left( \delta_{d,1}-\delta_{d,2} \right)^{2}+\left( \delta_{p,1}-\delta_{p,2} \right)^{2}+\left( \delta_{h,1}-\delta_{h,2} \right)^{2}} (13)$$

A smaller *R_a_* value indicates a shorter distance in Hansen space and thus a higher relative affinity between the two substances. Because the HSPs of solvent mixtures were estimated using a volume-fraction-weighted mixing rule, the resulting values should be regarded as approximate. Accordingly, the calculated Hansen distances were used mainly for qualitative or semi-quantitative comparison.

# Estimation of apparent relative polyamide crosslinking degree from XPS

The apparent relative crosslinking degree of the polyamide selective layer was estimated from the oxygen-to-nitrogen atomic ratio (O/N) obtained by XPS, following a previously reported method. The polyamide structure was approximated as a combination of fully crosslinked repeat units and linear repeat units, with fractions 𝑚 and 𝑛, respectively, satisfying

$$m+n=1 (14)$$

For this system, the fully crosslinked unit contains three oxygen atoms and three nitrogen atoms, whereas the linear unit contains four oxygen atoms and two nitrogen atoms. Therefore, the O/N atomic ratio can be expressed as

$$\frac{O}{N}=\frac{3m+4n}{3m+2n} (15)$$

The crosslinking degree was then estimated from the fitted values of *m* and *n*.

# Gravimetric measurement of toluene uptake

Free-standing MPD-TMC and TFMPD-TMC-6 PA layers were obtained by dissolving the PAN supports of the corresponding TFC membranes in DMAc. The isolated PA layers were thoroughly rinsed with deionized water to remove residual DMAc and then dried under vacuum at 100 °C until a constant mass was obtained. Approximately 40 mg of each dried PA sample was used for the swelling measurement. The initial dry mass, *m_dry_*, was recorded, after which the samples were immersed in toluene at room temperature. At 24 h intervals, the samples were removed from toluene and gently blotted with Kimwipes using an identical procedure to remove excess surface liquid. The samples were immediately weighed to obtain *m_wet_* and then re-immersed in fresh toluene. The gravimetric toluene uptake was calculated as follows^[10, 11]^:

$$Mass change (\%)=\left( \frac{m_{wet}-m_{dry}}{m_{dry}} \right)\times100\% (16)$$

# Molecular modeling and simulation

Crosslinked polyamide (PA) networks, including TFMPD-TMC and MPD-TMC systems, were constructed using the Amorphous Cell module in Materials Studio 2023. For both systems, the initial simulation box contained 110 TMC molecules and 150 diamine molecules, corresponding to 330 acyl chloride reaction sites from TMC and 300 amine reaction sites from MPD or TFMPD. Amide bonds were formed between TMC and MPD or TFMPD reactive sites to generate crosslinked PA networks. The final MPD-TMC network had a simulation-defined amine-site conversion of 90.0%, whereas the TFMPD-TMC network had a simulation-defined amine-site conversion of 87.7%.

For solvent-swollen simulations, toluene molecules were introduced into the equilibrated dry polyamide networks, followed by NPT equilibration to obtain the swollen membrane-solvent systems. After equilibration, the toluene number density in the swollen MPD-TMC network was approximately 1.4 molecules nm⁻³, whereas that in the swollen TFMPD-TMC network was approximately 0.6 molecules nm⁻³. The lower equilibrated toluene content in TFMPD-TMC is consistent with the reduced hydrocarbon uptake and smaller simulated volumetric swelling of the fluorinated polyamide network.

To describe electrostatic interactions, especially considering the electron-withdrawing effect of the –CF₃ group, atomic partial charges were calculated using xTB at the GFN1-xTB level with the CM5 charge model, and the obtained charges were assigned to the corresponding atoms in the PA network for subsequent molecular dynamics (MD) simulations. All MD simulations were performed using GROMACS 2020.6 with the General Amber Force Field (GAFF)^[12]^.

Force field parameterization of the –CF₃ group. The intramolecular parameters, including bond-stretching, angle-bending, and torsional potentials, and the intermolecular Lennard-Jones parameters for the –CF₃ group were adopted directly from the standard General AMBER Force Field (GAFF) database.^[12]^ Specifically, the fluorine atoms and the trifluoromethyl carbon were assigned the standard GAFF atom types f and c3, respectively. The GAFF parameters for halogenated organic compounds have been validated against quantum mechanical calculations and experimental condensed-phase thermodynamic properties.^[12]^ The molecular topology and corresponding force field parameters were generated using Sobtop, which assigned the standard GAFF parameters to the cross-linked polyamide network based on atomic connectivity and hybridization.^[13]^

Because the –CF₃ group is strongly electron-withdrawing, system-specific atomic partial charges were calculated rather than adopting standard or default empirical charges. Specifically, Charge Model 5 (CM5) atomic partial charges were calculated using the xTB software at the GFN1-xTB level.^[14]^ Therefore, the bonded and Lennard-Jones parameters of the –CF₃ group were obtained from the established GAFF database, whereas the electrostatic parameters were determined through quantum chemical calculations. No unvalidated custom force field parameters were introduced for the –CF₃ group.

The leap-frog integrator was used with a time step of 1 fs (dt = 0.001 ps). The dry polyamide networks were first subjected to energy minimization using the steepest descent algorithm and annealing to reduce local energy minima and promote chain relaxation, followed by a 20 ns NPT equilibration at 300 K and 1 atm to obtain equilibrated dry-state structures and densities.

For solvent-swollen systems, toluene molecules, parameterized with GAFF and CM5 charges consistent with the PA network, were inserted into the equilibrated dry networks using the *gmx solvate* tool while avoiding atomic overlap with the PA framework. A 50 ns NPT simulation was then conducted to equilibrate the membrane-solvent system until the box volume fluctuated within ±1% for at least 5 ns.

The simulation parameters were set as follows: electrostatic interactions were treated using the Particle Mesh Ewald (PME) method with a real-space cutoff of 1.2 nm, while van der Waals interactions were computed using a cutoff of 1.2 nm with long-range dispersion corrections applied to both energy and pressure (DispCorr = EnerPres) to improve the accuracy of density calculations for amorphous polymer systems. The temperature was controlled using the velocity-rescaling thermostat (v-rescale) with a coupling time constant (τ-T) of 0.1 ps, maintaining the system at 300 K throughout the equilibration and sampling periods. The pressure was maintained at 1 atm (1.01325 bar) using the Berendsen barostat under isotropic conditions, with a coupling time constant (τ-P) of 5.0 ps and a compressibility of 4.5 × 10⁻⁵ bar⁻¹. The Berendsen barostat was used for density equilibration and structural relaxation; therefore, the simulations were used primarily for comparative structural analysis rather than rigorous ensemble-property calculations.

All hydrogen bonds were constrained using the LINCS algorithm^[15]^, allowing for stable integration with a 1 fs time step. The neighbor list was updated every 20 steps using the Verlet cutoff scheme to optimize the computational efficiency of non-bonded interactions. After equilibration, trajectory files were converted to PDB and CIF formats for post-processing. The pore-size distribution (PSD) was analyzed using Zeo++, while the fractional free volume (FFV) was calculated for structural characterization, to compare the effect of the -CF₃ group on the structural evolution of polyamide networks under dry and swollen conditions.

Free-volume connectivity was evaluated using the Zeo++ Voronoi tessellation algorithm. The method abstracts the free-volume network into a graph composed of nodes (cavities) and edges (throats connecting adjacent cavities): a throat is marked as accessible when its radius exceeds the selected probe radius, and a percolating network is identified when accessible throats form a continuous pathway across periodic boundaries. In this work, probe radii of 0.5-0.8 Å were employed for dry networks, while radii of 0.7-1.0 Å were used for the toluene-swollen networks to capture percolation behavior in these ultramicroporous systems.

It should be noted that pore-size distribution characterizes the maximum dimensions of individual cavities, whereas connectivity is governed by the throats linking these cavities. In densely crosslinked glassy polymers, the throat dimensions are substantially smaller than the cavity dimensions, and the overall connectivity is limited by the narrowest throats. In addition, static Voronoi analysis is performed on configurations with fixed atomic positions and therefore does not capture thermal vibrations or segmental motions that dynamically generate transient free-volume elements under operating conditions. Consequently, the small probe radii used here should be interpreted as connectivity thresholds for probing the intrinsic free-volume skeleton of the crosslinked polymer networks, rather than as literal penetrant sizes. Applying this small-probe series to static snapshots enabled comparison of differences in intrinsic connectivity constraints imposed by the polymer backbones.

**Results and Discussion**

## Figure S1. Schematic of the interfacial polymerization systems. (a) MPD-TMC and (b) TFMPD-TMC, showing the monomers in each phase and the corresponding polyamide network structures. The network is represented as a combination of fully crosslinked (m) and linear (n) repeat units.

## Figure S2. Schematic of methanol-assisted interfacial polymerization on a PAN support.

## Figure S3. Conventional roll-to-roll interfacial polymerization process for polyamide thin-film composite membrane fabrication.

## Figure S4. Surface morphology of the PAN support.

## Figure S5. Cross-sectional SEM images of TFMPD-TMC membranes prepared with methanol contents of (a) 3, (b) 6, (c) 10, (d) 20, and (e) 46 wt.% in the aqueous phase.

## Figure S6. Intrinsic PA thicknesses of TFMPD-TMC membranes. Representative plan-view TEM images of isolated PA films prepared with (a) 3, (b) 6, (c) 10, and (d) 46 wt.% methanol. For each membrane, the mean intrinsic PA thickness (𝛿_int_) was calculated from 10 TEM images (*n*=10). The white markers indicate representative measurement positions. Regions containing evident intersections, folds, or overlapping PA films were excluded from the analysis. Data are presented as mean ± standard deviation.

## Figure S7. FTIR spectra of PAN, MPD-TMC, and TFMPD-TMC membranes.

Note: The pristine PAN substrate exhibits a characteristic absorption band at 2245 cm⁻¹, assigned to nitrile (C≡N) stretching. After interfacial polymerization, MPD-TMC and TFMPD-TMC membranes show amide bands at ~1670 cm⁻¹ (amide I, C=O stretching) and ~1550 cm⁻¹ (amide II, N–H bending/C–N stretching), consistent with formation of the polyamide selective layer. TFMPD-TMC membranes additionally exhibit a band at ~1120 cm⁻¹, assigned to C–F stretching of the trifluoromethyl group**^[16-18]^**.

## Figure S8. High-resolution XPS spectra of the TFMPD-TMC-3 membrane.

## Figure S9. High-resolution XPS spectra of the TFMPD-TMC-6 membrane.

## Figure S10. High-resolution XPS spectra of the TFMPD-TMC-10 membrane.

## Figure S11. High-resolution XPS spectra of the TFMPD-TMC-20 membrane.

## Figure S12. High-resolution XPS spectra of the TFMPD-TMC-46 membrane.

## Figure S13. High-resolution XPS spectra of the MPD-TMC membrane.

## Figure S14. XPS survey spectra of MPD-TMC and TFMPD-TMC-6 membranes.

**Note**: MPD-TMC shows the characteristic C 1s, N 1s, and O 1s signals of polyamide selective layers, whereas TFMPD-TMC-6 additionally exhibits an F 1s peak, indicating the incorporation of TFMPD-derived fluorinated moieties.

## Figure S15. Representative water contact-angle images of TFMPD-TMC membranes prepared with methanol concentrations of (a) 3, (b) 6, (c) 10, (d) 20, and (e) 46 wt.% in the aqueous phase. Quantitative values are summarized in Table S3.

## Figure S16. SEM-EDS elemental mapping of the MPD-TMC membrane.

## Figure S17. SEM-EDS elemental mapping of the TFMPD-TMC-6 membrane.

## Figure S18. Surface SEM images of (a) MPD-TMC and (b) TFMPD-TMC-6 membranes.

## Figure S19. Young's modulus maps of (a) MPD-TMC and (b) TFMPD-TMC-6 membranes obtained by atomic force microscopy in quantitative nanomechanical mapping (QNM) mode over a 5 × 5 µm scan area.

## Figure S20. TGA curves of (a) MPD-TMC and (b) TFMPD-TMC-6 polyamide powders. The degradation temperature, T_d_, defined at 5% weight loss, increased from 184 °C for MPD-TMC to 205 °C for TFMPD-TMC-6.

## Figure S21. Representative static contact-angle images of water, glycerol, and diiodomethane on (a) MPD-TMC and (b) TFMPD-TMC-6 membranes.

## Figure S22. Three-dimensional views of amorphous cells containing (a) MPD-TMC and (b) TFMPD-TMC polyamide networks. The light-blue surface represents the accessible surface calculated using a probe radius of 1 Å. Cell size: 40 Å × 40 Å × 40 Å. Atom color code: C, gray; O, red; N, blue; F, cyan; H, white.

## Figure S23. Computational modeling and fractional free-volume (FFV) snapshots of dry polyamide networks. (a) MPD-TMC and (b) TFMPD-TMC. Grey regions denote the polymer network, and blue regions denote the calculated free volume. Atom color code: C, gray; O, red; N, blue; F, cyan; H, white.

## Figure S24. Computational modeling and fractional free-volume (FFV) snapshots of toluene-swollen polyamide networks. (a) MPD-TMC and (b) TFMPD-TMC. Grey regions denote the polymer network, and blue regions denote the calculated free volume. Atom color code: C, gray; O, red; N, blue; F, cyan; H, white.

## Figure S25. Gravimetric toluene uptake of free-standing MPD-TMC and TFMPD-TMC-6 polyamide layers as a function of immersion time.

## Figure S26. Dry-state free-volume connectivity of MPD-TMC. (a-d) Zeo++ Voronoi-based percolation maps calculated using probe radii of 0.5, 0.6, 0.7, and 0.8 Å, respectively. Blue and red regions denote interconnected and disconnected voids, respectively.

## Figure S27. Dry-state free-volume connectivity of TFMPD-TMC. (a-d) Zeo++ Voronoi-based percolation maps calculated using probe radii of 0.5, 0.6, 0.7, and 0.8 Å, respectively. Blue and red regions denote interconnected and disconnected voids, respectively.

## Figure S28. Free-volume connectivity of toluene-swollen MPD-TMC. (a-d) Zeo++ Voronoi-based percolation maps calculated using probe radii of 0.7, 0.8, 0.9, and 1.0 Å, respectively. Blue and red regions denote interconnected and disconnected voids, respectively.

## Figure S29. Free-volume connectivity of toluene-swollen TFMPD-TMC. (a-d) Zeo++ Voronoi-based percolation maps calculated using probe radii of 0.7, 0.8, 0.9, and 1.0 Å, respectively. Blue and red regions denote interconnected and disconnected voids, respectively.

## Figure S30. Correlations between single-solvent permeance and solvent properties for TFMPD-TMC-6. Relationships between solvent permeance and (a) viscosity (*η*), (b) molecular diameter (*d_m_*), (c) Hansen polar parameter (*δ_p_*), and (d) a combined parameter accounting for viscosity and molecular diameter.

## Figure S31. Qualitative relationship between hydrocarbon rejection and an aromaticity/size descriptor.

Note: The descriptor, $(1+{{Carbon}_{aromatic}}/{{Carbon}_{total}})/MW$, was used as a simplified metric to capture the combined effects of sorption tendency and diffusional hindrance. The term$(1+{{Carbon}_{aromatic}}/{{Carbon}_{total}})$ reflects the degree of aromaticity and was used as an approximate indicator of membrane affinity, whereas MW was used as an approximate descriptor of steric hindrance. A higher value of this descriptor therefore suggests stronger partitioning and/or lower diffusion resistance, which can correspond to lower rejection**^[11, 19]^**. This descriptor is used only for qualitative interpretation.

**Figure S32**. Average performance of TFMPD-TMC-6 at day 21 in long term tests.

#### Table S1. Hansen solubility parameters of methanol/water mixtures and their Hansen distances (*R_a_*) from n-hexane.

| **Methanol concentration (wt.%)** | **δ_d_ (MPa^1/2^)** | **δ_p_ (MPa^1/2^)** | **δ_h_ (MPa^1/2^)** | **R_a_ (MPa^1/2^)** |
| --- | --- | --- | --- | --- |
| 3 | 15.48 | 15.86 | 41.53 | 44.47 |
| 6 | 15.47 | 15.72 | 40.78 | 43.72 |
| 10 | 15.45 | 15.54 | 39.79 | 42.73 |
| 20 | 15.40 | 15.10 | 37.41 | 40.35 |
| 46 | 15.29 | 14.05 | 31.75 | 34.73 |

Note: The decrease in *R_a_* with increasing methanol concentration is primarily driven by reductions in the polar (*δ_p_*) and hydrogen-bonding (*δ_h_*) components of the aqueous phase, whereas the dispersive component (*δ_d_*) changes only slightly.

#### Table S2. XPS elemental composition and apparent relative crosslinking degree of polyamide TFC membranes.

| **Membranes** | **C (%)** | | **N (%)** | **O (%)** | **F (%)** | **O/N** | **Crosslinking degree (%)** |
| --- | --- | --- | --- | --- | --- | --- | --- |
| MPD-TMC | | 73.44 | 11.11 | 15.44 | - | 1.39 | 51 |
| TFMPD-TMC-3 | | 66.66 | 8.86 | 13.23 | 11.26 | 1.49 | 41 |
| TFMPD-TMC-6 | | 67.43 | 9.35 | 12.61 | 10.63 | 1.35 | 55 |
| TFMPD-TMC-10 | | 67.94 | 9.43 | 13.19 | 9.44 | 1.40 | 50 |
| TFMPD-TMC-20 | | 67.82 | 9.05 | 13.43 | 9.70 | 1.48 | 42 |
| TFMPD-TMC-46 | | 66.54 | 8.67 | 13.65 | 11.15 | 1.57 | 33 |

#### Table S3. Water contact angles and surface roughness of TFMPD-TMC membranes prepared with different methanol concentrations.

| **Methanol concentration (wt.%)** | **Water contact angle (°)** | **Surface roughness (nm)** |
| --- | --- | --- |
| 3 | 107.4 ± 1.0 | 53.6 ± 11.0 |
| 6 | 111.3 ± 5.2 | 62.7 ± 4.8 |
| 10 | 114.3 ± 5.2 | 60.5 ± 7.9 |
| 20 | 108.0 ± 5.9 | 61.5 ± 6.2 |
| 46 | 89.8 ± 4.8 | 23.6 ± 0.6 |

#### Table S4. Toluene/TIPB separation performance of membranes prepared with different methanol concentrations.

| **Membrane** | **Methanol concentration (wt.%)** | **Toluene permeance**  **(L m^-2^ h^-1^ bar^-1^)** | **TIPB rejection (%)** |
| --- | --- | --- | --- |
| MPD-TMC | 0 | 0.019 ± 0.001 | 5.94 ± 7.54 |
| TFMPD-TMC-3 | 3 | 0.099 ± 0.024 | 85.53 ± 0.93 |
| TFMPD-TMC-6 | 6 | 0.227 ± 0.049 | 98.11 ± 0.50 |
| TFMPD-TMC-10 | 10 | 0.166 ± 0.039 | 90.98 ± 0.88 |
| TFMPD-TMC-20 | 20 | 0.091 ± 0.013 | 75.91 ± 4.19 |
| TFMPD-TMC-46 | 46 | 0.068 ± 0.019 | 54.62 ± 4.15 |

Note: Measurements were performed using a 99/1 mol% toluene/TIPB feed at room temperature and 20 bar.

#### Table S5. Static contact angles of probe liquids on MPD-TMC and TFMPD-TMC-6 membranes.

| **Membrane** | **Water (°)** | **Glycerol (°)** | **Diiodomethane (°)** |
| --- | --- | --- | --- |
| MPD-TMC | 56.3 ± 2.5 | 43.8 ± 0.5 | 9.5 ± 1.5 |
| TFMPD-TMC-6 | 111.3 ± 5.2 | 85.7 ± 2.8 | 71.1 ± 2.8 |

#### Table S6. Surface-tension components of probe liquids used for surface free-energy calculations at 20 °C. Values are given in mN m^-1^.

| **Liquid** | **γ^LW^** | **γ^+^** | **γ^−^** | **γ^AB^** | **γ^total^** |
| --- | --- | --- | --- | --- | --- |
| Water | 21.8 | 25.5 | 25.5 | 51.0 | 72.8 |
| Glycerol | 34.0 | 3.9 | 57.4 | 30.0 | 64.0 |
| Diiodomethane | 50.8 | 0 | 0 | 0 | 50.8 |

Note: Diiodomethane has zero electron-acceptor and electron-donor components and was therefore used to determine the Lifshitz–van der Waals component of the membrane surface free energy. Water and glycerol have distinct electron-acceptor and electron-donor components, and their contact-angle data were jointly used to determine the corresponding electron-acceptor and electron-donor components of the membrane surface. The acid–base component and total surface free energy were subsequently calculated from these components.

#### Table S7. Surface free-energy parameters of MPD-TMC and TFMPD-TMC-6 membranes. Values are given in mN m^-1^.

| **Membrane** | **γ^total^** | **γ^LW^** | **γ^AB^** | **γ^+^** | **γ^−^** |
| --- | --- | --- | --- | --- | --- |
| MPD-TMC | 56.4 | 50.1 | 6.3 | 0.7 | 14.8 |
| TFMPD-TMC-6 | 24.4 | 22.3 | 2.1 | 1.3 | 0.9 |

#### Table S8. Comparison of volumetric swelling of MPD-TMC and TFMPD-TMC after toluene equilibration.

|  | **MPD-TMC** | | **TFMPD-TMC** |
| --- | --- | --- | --- |
| Dry-state volume (10⁻³ nm³) | | 89716.8 | 98272.1 |
| Toluene-swollen volume (10⁻³ nm³) | | 108275.0 | 103528.0 |
| Volumetric swelling ratio (%) | | 20.69 | 5.35 |

Note: The dry and toluene-swollen simulation cell volumes are reported in units of 10⁻³ nm³. The volumetric swelling ratio was calculated as:

$$Swelling ratio=\frac{V_{swollen}-V_{\mathrm{dry}}}{V_{\mathrm{dry}}}\times100\%$$

#### Table S9. Comparison of toluene/TIPB separation performance for membranes in this work and representative literature membranes.

| **Membrane** | **Toluene Permeance (L m⁻² h⁻¹ bar⁻¹)** | **Toluene/TIPB separation factor** | **Applied pressure (bar)** | **TIPB**  **concentration**  **(mol%)** | **Ref.** |
| --- | --- | --- | --- | --- | --- |
| **MPD-TMC** | 0.019 ± 0.001 | 1.07 ± 0.08 | 20 | 1 | **This work** |
| **TFMPD-TMC-3** | 0.099 ± 0.024 | 6.98 ± 0.45 | 20 | 1 |  |
| **TFMPD-TMC-6** | 0.227 ± 0.049 | 55.09 ± 14.50 | 20 | 1 |  |
| **TFMPD-TMC-10** | 0.166 ± 0.039 | 11.25 ± 1.05 | 20 | 1 |  |
| **TFMPD-TMC-20** | 0.091 ± 0.013 | 4.28 ± 0.74 | 20 | 1 |  |
| **TFMPD-TMC-46** | 0.068 ± 0.019 | 2.23 ± 0.19 | 20 | 1 |  |
| Torlon hollow fiber | 0.01 | 12 | 60 | 1 | ^[20]^ |
| Crosslinked ASPOC MMCM TFC-9.4 vol% | 0.42 | 3.95 | 30 | 5 | ^[21]^ |
| Diamine Crosslinked Matrimid hollow fiber | 0.0059 | 9.9 | 10 | 5 |  |
| UIO-66/Matrimid MMM hollow fiber | 0.033 | 5.9 | 10 | 5 |  |
| CuAAC Crosslinked Matrimid TFC | 2.045 | 1.07 | 30 | 5 |  |
| Crosslinked ASPOC MMCM TFC-5.7 vol% | 0.51 | 3.72 | 30 | 5 |  |
| Crosslinked ASPOC MMCM TFC-2.6 vol% | 0.17 | 9.67 | 30 | 5 |  |
| PIM-1 | 3.5 | 1.11 | 15 | 1 | ^[11]^ |
| SBAD-1 | 0.2 | 5.20 | 15 | 1 |  |
| SBAD-2 | 0.19 | 3.70 | 15 | 1 |  |
| SBAD-3 | 0.7 | 3.13 | 15 | 1 |  |
| SBAD-4 | 0.26 | 1.67 | 15 | 1 |  |
| AlOx/PIM-1 | 1.2 | 14 | 30 | 2.32 | ^[22]^ |
| Fluorinated polyamide | 0.15 | 18 | 30 | 2.32 | ^[23]^ |
| SBAD-1 | 0.03 | 7.52 | 10 | 1 | ^[24]^ |
| SBAD-1-Me | 0.052 | 2.96 | 10 | 1 |  |
| SBAD-1-Bz | 0.10436 | 4.54 | 10 | 1 |  |
| SBAD-1-FBz | 0.29 | 2.05 | 10 | 1 |  |
| PIM-1 | 1.07 | 1.53 | 10 | 1 |  |
| SBAD-5 | 0.21 | 3.63 | 10 | 1 |  |
| SBAD-6 | 0.12 | 3.97 | 10 | 1 |  |
| SBAD-7 | 0.042 | 3.43 | 10 | 1 |  |
| SBiAD-1 | 0.099 | 2.03 | 10 | 1 |  |
| SBiAD-2 | 1.02 | 1.16 | 10 | 1 |  |
| PIM-SBF | 0.17 | 2.08 | 10 | 1 |  |
| Ducky-9 | 0.23 | 2.43 | 30 | 1 | ^[19]^ |
| Ducky-10 | 0.31 | 1.52 | 30 | 1 |  |
| Matrimid | 0.35 | 2.46 | 30 | 10 | ^[25]^ |
| Solid-State Crosslinked Matrimid | 0.21 | 10.32 | 30 | 10 |  |
| Solid-State Crosslinked Matrimid | 0.16 | 10.19 | 30 | 10 |  |
| Solid-State Crosslinked Matrimid | 0.12 | 10.04 | 30 | 10 |  |
| Puramem Selective | 0.66 | 1.78 | 30 | 10 |  |
| Polyimide P84 with graft polymerization | 0.21 | 6.1 | 45 | 5 | ^[26]^ |
| Polyimide P84 with graft polymerization | 0.33 | 1.2 | 45 | 5 |  |
| Polyimide P84 with graft polymerization | 0.36 | 2.9 | 45 | 5 |  |
| Polyimide P84 with graft polymerization | 0.39 | 1.1 | 45 | 5 |  |
| MPD-SBF | 0.247 | 3.95 | 50 | 2 | ^[27]^ |
| PPD-TMC | 0.415 | 3.3 | 50 | 2 |  |
| PPD-SBF | 2.49 | 3.83 | 50 | 2 |  |
| PIP-TMC | 0.283 | 2.57 | 50 | 2 |  |
| PIP-SBF | 0.143 | 2.78 | 50 | 2 |  |
| MPD-SBF | 0.089 | 2.8 | 50 | 2 | ^[28]^ |
| MPD-TFB | 0.017 | 2.4 | 50 | 2 |  |
| MPD-TFS | 0.22 | 5.6 | 50 | 2 |  |
| Trip-TFB | 0.11 | 30.7 | 50 | 2 |  |
| Trip-TFS | 0.31 | 19.3 | 50 | 2 |  |
| Puramem | 0.72 | 1.8 | 50 | 2 |  |
| FRPAA-1 | 0.33 | 9.76 | 50 | 1 | ^[29]^ |
| FRPAA-2 | 0.23 | 8.5 | 50 | 1 |  |
| FRPAA-3 | 0.40 | 3.71 | 50 | 1 |  |
| FRPAA-4 | 0.52 | 4.93 | 50 | 1 |  |
| A4M0-M | 0.57 | 7.21 | 8 | 1 | ^[30]^ |
| A3M1-M | 1.26 | 3.67 | 8 | 1 |  |
| A2M2-M | 3.55 | 2.15 | 8 | 1 |  |
| A1M3-M | 4.90 | 1.69 | 8 | 1 |  |

Note: The literature data were obtained under different operating conditions, including applied pressures of 8-60 bar and TIPB concentrations of 1-10 mol%. The TFMPD-TMC-6 membrane in this work was evaluated using a 99/1 mol% toluene/TIPB feed at room temperature and 20 bar. The comparison is therefore intended to illustrate the broader OSRO performance landscape rather than a direct ranking under identical conditions.

#### Table S10. Long-term toluene/TIPB separation performance of TFMPD-TMC-6 for a toluene/TIPB binary mixture (99/1 mol%) at room temperature and 20 bar.

| **Time (day)** | **Toluene permeance (L m^-2^ h^-1^ bar^-1^)** | **TIPB rejection (%)** |
| --- | --- | --- |
| 0.52 | 0.28 | 96.31 |
| 0.87 | 0.29 | 96.65 |
| 0.92 | 0.33 | 96.91 |
| 1.40 | 0.28 | 97.04 |
| 1.62 | 0.30 | 97.24 |
| 1.93 | 0.27 | 97.07 |
| 2.39 | 0.27 | 97.20 |
| 2.63 | 0.27 | 97.64 |
| 2.87 | 0.24 | 96.90 |
| 3.25 | 0.23 | 96.45 |
| 3.87 | 0.20 | 96.91 |
| 4.32 | 0.20 | 97.36 |
| 4.87 | 0.17 | 97.73 |
| 5.34 | 0.16 | 97.69 |
| 5.88 | 0.14 | 97.50 |
| 6.29 | 0.13 | 97.58 |
| 6.90 | 0.14 | 97.72 |
| 7.85 | 0.14 | 97.61 |
| 8.37 | 0.15 | 97.48 |
| 8.88 | 0.14 | 97.39 |
| 9.35 | 0.14 | 97.44 |
| 9.85 | 0.14 | 97.51 |
| 10.39 | 0.15 | 97.05 |
| 11.05 | 0.12 | 97.33 |
| 12.01 | 0.09 | 97.75 |
| 12.89 | 0.10 | 97.63 |
| 14.07 | 0.10 | 97.63 |
| 15.24 | 0.10 | 97.65 |
| 16.20 | 0.10 | 97.54 |
| 17.22 | 0.10 | 97.55 |
| 18.18 | 0.10 | 97.43 |
| 19.12 | 0.10 | 97.38 |
| 19.96 | 0.11 | 97.40 |
| 21.17 | 0.10 | 97.05 |

#### Table S11. Single-solvent permeance of TFMPD-TMC-6.

| **Solvents** | **Permeance (L m^-2^ h^-1^ bar^-1^)** |
| --- | --- |
| 1-Hexene | 0.59 ± 0.048 |
| Toluene | 0.51 ± 0.051 |
| n-Hexane | 0.22 ± 0.023 |
| n-Heptane | 0.15 ± 0.003 |

Note: Measurements were performed at room temperature and 20 bar.

#### Table S12. Hansen solubility parameters and physical properties of the solvents used in this study^[7, 31-33]^.

| **Solvent** | **𝑑_𝑚_ (nm)** | **Viscosity (η) at 30 ℃ (mPa·s)** | **δ_d_ (MPa^1/2^)** | **δ_p_ (MPa^1/2^)** | **δ_h_ (MPa^1/2^)** | **δ (MPa^1/2^)** |
| --- | --- | --- | --- | --- | --- | --- |
| Water | 0.39 | 0.80 | 15.5 | 16.0 | 42.3 | 47.8 |
| Methanol | 0.51 | 0.49 | 15.1 | 12.3 | 22.3 | 29.6 |
| n-Hexane | 0.75 | 0.28 | 14.9 | 0 | 0 | 14.9 |
| 1-Hexene | 0.74 | 0.26 | 14.7 | 0 | 0 | 14.7 |
| n-Heptane | 0.78 | 0.37 | 15.3 | 0 | 0.0 | 15.3 |
| Toluene | 0.70 | 0.52 | 18.0 | 1.4 | 2.0 | 18.2 |

Note: 𝑑_𝑚_ denotes the approximate molecular diameter. It was estimated from the molar volume (*V_m_*) of each solvent molecule by assuming an equivalent spherical volume: $d_{m}=2\times{({3V_{m}}/{4\pi N_{A}})}^{1/3}$; where *N_A_* is the Avogadro's constant^[34]^.

#### Table S13. Hydrocarbon rejection of the TFMPD-TMC-6 membrane determined from permeation tests using a dilute 15-solute feed mixture (1 mol% each solute in toluene) at room temperature and 20 bar.

| **Component** | **Molecular weight (g mol^-1^)** | **Rejection (%)** |
| --- | --- | --- |
| Isooctane | 114.22 | 68.45 ± 6.16 |
| n-Octane | 114.22 | 23.11 ± 1.95 |
| p-Xylene | 106.16 | -3.88 ± 2.35 |
| o-Xylene | 106.16 | 0.75 ± 2.41 |
| Propylcyclohexane | 126.24 | 47.20 ± 3.43 |
| Propylbenzene | 120.19 | 0.61 ± 3.03 |
| Mesitylene | 120.19 | 21.83 ± 3.08 |
| Decalin | 138.25 | 66.75 ± 4.04 |
| Naphthalene | 128.17 | -6.53 ± 3.61 |
| Dodecane | 170.33 | 57.94 ± 2.66 |
| 1-Methylnaphthalene | 142.20 | 13.04 ± 2.85 |
| TIPB | 204.35 | 95.26 ± 1.06 |
| Biphenyl | 154.21 | -5.48 ± 3.75 |
| Dodecylbenzene | 246.43 | 80.30 ± 1.18 |
| n-Docosane | 310.60 | 94.56 ± 0.48 |

#### Table S14. Feed composition of the model Fischer–Tropsch hydrocarbon mixture used in permeation experiments.

| **Hydrocarbon** | **Molecular weight (g mol^-1^)** | **Mass fraction (wt.%)** |
| --- | --- | --- |
| 1-Hexene | 84.16 | 3.90 |
| n-C6 (n-Hexane) | 86.18 | 21.10 |
| n-C7 (n-Heptane) | 100.21 | 16.70 |
| n-C8 (n-Octane) | 114.22 | 18.20 |
| n-C12 (Dodecane) | 170.33 | 10.40 |
| n-C16 (Hexadecane) | 226.41 | 10.70 |
| n-C18 (Octadecane) | 254.49 | 11.20 |
| n-C22 (Docosane) | 310.60 | 7.82 |

#### Table S15. Separation performance of TFMPD-TMC-6 for the model Fischer–Tropsch feed at room temperature and 35 bar.

| **Hydrocarbon** | **C_P_/C_R_** |
| --- | --- |
| 1-Hexene | 1.47 ± 0.02 |
| n-C6 (n-Hexane) | 1.31 ± 0.01 |
| n-C7 (n-Heptane) | 1.07 ± 0.08 |
| n-C8 (n-Octane) | 0.95 ± 0.01 |
| n-C12 (Dodecane) | 0.69 ± 0.02 |
| n-C16 (Hexadecane) | 0.45 ± 0.03 |
| n-C18 (Octadecane) | 0.34 ± 0.03 |
| n-C22 (Docosane) | 0.19 ± 0.04 |
| Permeance (L m^-2^ h^-1^ bar^-1^) | 0.017 ± 0.004 |

#### Table S16. Olefin/paraffin separation performance of TFMPD-TMC-6 for feed mixtures with different 1-hexene contents at room temperature and 30 bar.

| **1-Hexene content in feed** | **Component** | **Component mass fraction (wt.%)** | **Rejection (%)** | **Total permeance**  **(L m^-2^ h^-1^ bar^-1^)** |
| --- | --- | --- | --- | --- |
| 75 wt.% | 1-Hexene | 75.00 | -12.17 ± 3.53 | 0.074 ± 0.007 |
|  | n-Hexane  (n-C6) | 3.125 | -0.47 ± 0.20 |  |
|  | 2,2-Dimethylbutane (2,2-DMB) | 3.125 | 61.80 ± 1.40 |  |
|  | n-Octane  (n-C8) | 8.75 | 20.04 ± 5.79 |  |
|  | Isooctane  (iso-C8) | 3.75 | 66.71 ± 2.81 |  |
|  | n-Dodecane  (n-C12) | 6.25 | 50.54 ± 6.69 |  |
| 84 wt.% | 1-Hexene | 84.00 | -8.45 ± 0.05 | 0.115 ± 0.034 |
|  | n-Hexane  (n-C6) | 2.00 | 5.16 ± 0.84 |  |
|  | 2,2-Dimethylbutane (2,2-DMB) | 2.00 | 59.74 ± 1.43 |  |
|  | n-Octane  (n-C8) | 5.60 | 24.23 ± 4.11 |  |
|  | Isooctane  (iso-C8) | 2.40 | 68.93 ± 0.22 |  |
|  | n-Dodecane  (n-C12) | 4.00 | 55.15 ± 1.09 |  |
| 90 wt.% | 1-Hexene | 90.00 | -4.62 ± 0.13 | 0.163 ± 0.028 |
|  | n-Hexane  (n-C6) | 1.25 | 7.16 ± 0.31 |  |
|  | 2,2-Dimethylbutane (2,2-DMB) | 1.25 | 65.81 ± 7.33 |  |
|  | n-Octane  (n-C8) | 3.50 | 28.03 ± 0.79 |  |
|  | Isooctane  (iso-C8) | 1.50 | 75.29 ± 3.38 |  |
|  | n-Dodecane  (n-C12) | 2.50 | 57.87 ± 1.09 |  |

#### Table S17. Experimentally measured stream compositions during three-stage membrane-cascade enrichment of 1-hexene.

| **Component** | **Feed (wt.%)** | **Stage 1 Permeate (wt.%)** | **Stage 2 Permeate (wt.%)** | **Stage 3 Permeate (wt.%)** |
| --- | --- | --- | --- | --- |
| Olefin | | | | |
| 1-Hexene | 75.00 | 84.72 ± 1.92 | 89.48 ± 0.15 | 92.71 ± 0.19 |
| Paraffins | | | | |
| n-Hexane  (n-C6) | 3.125 | 3.16 ± 0.03 | 3.01 ± 0.03 | 2.82 ± 0.08 |
| 2,2-Dimethylbutane (2,2-DMB) | 3.125 | 1.20 ± 0.05 | 0.51 ± 0.04 | 0.18 ± 0.05 |
| n-Octane  (n-C8) | 8.75 | 6.55 ± 1.47 | 5.16 ± 0.13 | 3.59 ± 0.11 |
| Isooctane  (iso-C8) | 3.75 | 1.26 ± 0.10 | 0.41 ± 0.05 | 0.12 ± 0.03 |
| n-Dodecane  (n-C12) | 6.25 | 3.12 ± 0.45 | 1.44 ± 0.10 | 0.58 ± 0.15 |
| Permeance  (L m^-2^ h^-1^ bar^-1^) | - | 0.074 ± 0.007 | 0.120 ± 0.024 | 0.149 ± 0.009 |

Note: Measurements were performed at room temperature and 30 bar using an initial feed containing 75 wt.% 1-hexene. The permeate from each stage was used as the feed for the subsequent stage. Stream compositions were normalized to 100 wt.% for reporting.

#### Table S18. Separation performance of TFMPD-TMC-6 for binary linear/branched alkane mixtures at room temperature and 30 bar.

| **Linear/branched alkanes** | **Linear permeance**  **(L m^-2^ h^-1^ bar^-1^)** | **Branched alkane rejection (%)** |
| --- | --- | --- |
| n-C6 / 2,2-DMB | 0.082 ± 0.018 | 56.09 ± 5.99 |
| n-C6 / 2,4-DMP | 0.060 ± 0.005 | 50.82 ± 0.05 |
| n-C6 / iso-C8 | 0.117 ± 0.019 | 62.95 ± 4.51 |
| n-C7 / iso-C8 | 0.070 ± 0.001 | 51.36 ± 3.08 |
| n-C8 / iso-C8 | 0.045 ± 0.012 | 49.97 ± 1.15 |

Note: The feed contained 99 mol% linear alkane and 1 mol% branched alkane. Abbreviations: n-C6, n-hexane; 2,2-DMB, 2,2-dimethylbutane; 2,4-DMP, 2,4-dimethylpentane; iso-C8, isooctane; n-C7, n-heptane; n-C8, n-octane.

#### Table S19. Pressure-dependent separation performance of TFMPD-TMC-6 for a 99/1 mol% n-heptane/isooctane feed at room temperature.

| **Pressure (bar)** | **Permeance**  **(L m^-2^ h^-1^ bar^-1^)** | **Flux**  **(L m^-2^ h^-1^)** | **Isooctane rejection (%)** |
| --- | --- | --- | --- |
| 20 | 0.067 ± 0.014 | 1.35 ± 0.29 | 48.29 ± 0.60 |
| 25 | 0.067 ± 0.005 | 1.69 ± 0.13 | 50.02 ± 2.62 |
| 30 | 0.070 ± 0.001 | 2.09 ± 0.03 | 51.36 ± 3.08 |
| 35 | 0.069 ± 0.009 | 2.41 ± 0.32 | 55.27 ± 3.83 |

Note: The pressure-dependent separation test was conducted using a 99/1 mol% n-heptane/isooctane mixture at room temperature. The membrane was first operated at 35 bar, followed by sequential measurements at 30, 25, and 20 bar. Therefore, the data at 20 bar were collected after the membrane had been exposed to the maximum investigated pressure of 35 bar.

**SI References**

[1]	M. F. Jimenez-Solomon, Q. Song, K. E. Jelfs, M. Munoz-Ibanez, A. G. Livingston, "Polymer nanofilms with enhanced microporosity by interfacial polymerization" *Nature Materials* **15**, (2016): 760-767.

[2]	Z. Zhou, Q. Wang, Y. Qin, Y. Hu, "Internal Concentration Polarization in the Polyamide Active Layer of Thin-Film Composite Membranes" *Environmental Science & Technology* **57**, (2023): 5999-6007.

[3]	L.-H. Lee, "Roles of molecular interactions in adhesion, adsorption, contact angle and wettability" *Journal of Adhesion Science and Technology* **7**, (1993): 583-634.

[4]	S. Yariv, H. Cross in Geochemistry of Colloid Systems: For Earth Scientists, Vol. (Eds.: Yariv, S., Cross, H.), Springer Berlin Heidelberg, Berlin, Heidelberg, (1979), pp. 93-155.

[5]	C. J. Van Oss, M. K. Chaudhury, R. J. Good, "Interfacial Lifshitz-van der Waals and polar interactions in macroscopic systems" *Chemical Reviews* **88**, (1988): 927-941.

[6]	C. J. van Oss, R. J. Good, M. K. Chaudhury, "Additive and nonadditive surface tension components and the interpretation of contact angles" *Langmuir* **4**, (1988): 884-891.

[7]	C. M. Hansen, Hansen Solubility Parameters: A User's Handbook, 2nd ed., CRC Press, Boca Raton, (2007).

[8]	X. Jiang, Y. Wang, M. Li, "Selecting water-alcohol mixed solvent for synthesis of polydopamine nano-spheres using solubility parameter" *Scientific Reports* **4**, (2014): 6070.

[9]	B. Khorshidi, B. Soltannia, T. Thundat, M. Sadrzadeh, "Synthesis of thin film composite polyamide membranes: Effect of monohydric and polyhydric alcohol additives in aqueous solution" *Journal of Membrane Science* **523**, (2017): 336-345.

[10]	Y. Ren, H. Ma, J. Kim, et al., "Fluorine-rich poly(arylene amine) membranes for the separation of liquid aliphatic compounds" *Science* **387**, (2025): 208-214.

[11]	K. A. Thompson, R. Mathias, D. Kim, et al., "N-Aryl–linked spirocyclic polymers for membrane separations of complex hydrocarbon mixtures" *Science* **369**, (2020): 310-315.

[12]	J. Wang, R. M. Wolf, J. W. Caldwell, P. A. Kollman, D. A. Case, "Development and testing of a general amber force field" *Journal of Computational Chemistry* **25**, (2004): 1157-1174.

[13]	S. Tian Lu,"Version 1.0(dev5),"http://sobereva.com/soft/Sobtop(accessed July 21, 2026).

[14]	A. V. Marenich, S. V. Jerome, C. J. Cramer, D. G. Truhlar, "Charge Model 5: An Extension of Hirshfeld Population Analysis for the Accurate Description of Molecular Interactions in Gaseous and Condensed Phases" *Journal of Chemical Theory and Computation* **8**, (2012): 527-541.

[15]	B. Hess, H. Bekker, H. J. C. Berendsen, J. G. E. M. Fraaije, "LINCS: A linear constraint solver for molecular simulations" *Journal of Computational Chemistry* **18**, (1997): 1463-1472.

[16]	C. Y. Tang, Y.-N. Kwon, J. O. Leckie, "Effect of membrane chemistry and coating layer on physiochemical properties of thin film composite polyamide RO and NF membranes: I. FTIR and XPS characterization of polyamide and coating layer chemistry" *Desalination* **242**, (2009): 149-167.

[17]	X.-L. Wang, Q.-H. Chen, Q.-H. Ye, et al., "Fully aromatic fluorinated polyamide nanofiltration membranes formed at gas/liquid interfaces with ultrafast nonpolar organic solvent permeance" *Journal of Membrane Science* **749**, (2026): 125427.

[18]	F. Alduraiei, P. Manchanda, B. Pulido, G. Szekely, S. P. Nunes, "Fluorinated thin-film composite membranes for nonpolar organic solvent nanofiltration" *Separation and Purification Technology* **279**, (2021): 119777.

[19]	N. C. Bruno, R. Mathias, Y. J. Lee, et al., "Solution-processable polytriazoles from spirocyclic monomers for membrane-based hydrocarbon separations" *Nature Materials* **22**, (2023): 1540-1547.

[20]	H.-Y. Jang, J. R. Johnson, Y. Ma, R. Mathias, D. A. Bhandari, R. P. Lively, "Torlon® hollow fiber membranes for organic solvent reverse osmosis separation of complex aromatic hydrocarbon mixtures" *AIChE Journal* **65**, (2019): e16757.

[21]	M. P. Rivera, N. C. Bruno, M. G. Finn, R. P. Lively, "Organic solvent reverse osmosis using CuAAC-crosslinked molecularly-mixed composite membranes" *Journal of Membrane Science* **638**, (2021): 119700.

[22]	E. K. McGuinness, F. Zhang, Y. Ma, R. P. Lively, M. D. Losego, "Vapor Phase Infiltration of Metal Oxides into Nanoporous Polymers for Organic Solvent Separation Membranes" *Chemistry of Materials* **31**, (2019): 5509-5518.

[23]	W. Kushida, R. R. Gonzales, T. Shintani, et al., "Organic solvent mixture separation using fluorine-incorporated thin film composite reverse osmosis membrane" *Journal of Materials Chemistry A* **10**, (2022): 4146-4156.

[24]	K. A. Thompson, R. Mathias, R. P. Lively, M. G. Finn, "Structure–Function Relationships in Membrane-Based Hydrocarbon Separations Using N-Aryl-Linked Spirocyclic Polymers" *Chemistry of Materials* **35**, (2023): 3464-3469.

[25]	Y. Feliachi, A. Roy, Y. Ren, M. G. Finn, R. P. Lively, "Solid-state crosslinking of thin film composite membranes for organic solvent reverse osmosis separations" *Journal of Membrane Science* **695**, (2024): 122462.

[26]	P. Ramesh, M. M. Sta Cruz, S. Karla, et al., "A new class of “structure-by-design” polymer membranes for organic solvent nanofiltration with controllable selectivity" *Journal of Membrane Science* **692**, (2024): 122296.

[27]	T. H. Lee, M. Balcik, W.-N. Wu, I. Pinnau, Z. P. Smith, "Dual-phase microporous polymer nanofilms by interfacial polymerization for ultrafast molecular separation" *Science Advances* **10**, (2024): eadp6666.

[28]	T. H. Lee, M. Balcik, Z. Ali, et al., "Microporous polyimine membranes for efficient separation of liquid hydrocarbon mixtures" *Science* **388**, (2025): 839-844.

[29]	Y. Ren, H. Ma, J. Kim, et al., "Fluorine-rich poly(arylene amine) membranes for the separation of liquid aliphatic compounds" *Science* **387**, (2025): 208-214.

[30]	W. Feng, F. Li, J. Li, et al., "Asymmetrical polyimide membranes with programmable polymer chain architectures for liquid hydrocarbon fractionation" *Science Advances* **11**, (2025): eady3674.

[31]	D. I. Sagdeev, M. G. Fomina, G. K. Mukhamedzyanov, I. M. Abdulagatov, "Experimental Study and Correlation Models of the Density and Viscosity of 1-Hexene and 1-Heptene at Temperatures from (298 to 473) K and Pressures up to 245 MPa" *Journal of Chemical & Engineering Data* **59**, (2014): 1105-1119.

[32]	S. Karan, Z. Jiang, A. G. Livingston, "Sub–10 nm polyamide nanofilms with ultrafast solvent transport for molecular separation" *Science* **348**, (2015): 1347-1351.

[33]	T. M. Aminabhavi, V. B. Patil, M. I. Aralaguppi, H. T. S. Phayde, "Density, Viscosity, and Refractive Index of the Binary Mixtures of Cyclohexane with Hexane, Heptane, Octane, Nonane, and Decane at (298.15, 303.15, and 308.15) K" *Journal of Chemical & Engineering Data* **41**, (1996): 521-525.

[34]	B. Van der Bruggen, J. Schaep, D. Wilms, C. Vandecasteele, "Influence of molecular size, polarity and charge on the retention of organic molecules by nanofiltration" *Journal of Membrane Science* **156**, (1999): 29-41.
